# KDM6A-ARHGDIB axis blocks metastasis of bladder cancer by inhibiting Rac1

**DOI:** 10.1186/s12943-021-01369-9

**Published:** 2021-05-18

**Authors:** Lei Liu, Jianfeng Cui, Yajing Zhao, Xiaochen Liu, Lipeng Chen, Yangyang Xia, Yong Wang, Shouzhen Chen, Shuna Sun, Benkang Shi, Yongxin Zou

**Affiliations:** 1 Department of Urology, Qilu Hospital, Cheeloo College of Medicine, Shandong University, Jinan, China; 2 Key Laboratory for Experimental Teratology of Ministry of Education, Department of Medical Genetics, School of Basic Medical Sciences, Shandong University, Jinan, Shandong China; 3 Key Laboratory of Urinary Precision Diagnosis and Treatment in Universities of Shandong, Jinan, China; 4 Department of Hematology, Qilu Hospital, Cheeloo College of Medicine, Shandong University, Jinan, China; 5 Department of Dermatology, The Affiliated Hospital of Shandong University of Traditional Chinese Medicine, Shandong Provincial Hospital of Traditional Chinese Medicine, Jinan, China

**Keywords:** Bladder cancer, Metastasis, Epigenetics, KDM6A, ARHGDIB-Rac1 axis, FOXA1

## Abstract

**Background:**

KDM6A, a histone demethylase, is frequently mutated in bladder cancer (BCa). However, the role and detailed molecular mechanism of KDM6A involved in bladder cancer progression remains unknown.

**Methods:**

Tissue specimens were used to determine the expression levels and prognostic values of KDM6A and ARHGDIB. The MTT, colony formation, wound healing and Transwell migration and invasion assays were employed to detect the BCa cell proliferation, migration and invasion, respectively. Chemotaxis of macrophages was used to evaluate the ability of KDM6A to recruit macrophages. A subcutaneous tumour model and tail vein tumour injection in nude mice were used to assess the role of KDM6A in vivo. RNA sequencing, qPCR, Western blot, ChIP and phalloidin staining assay were performed to investigate the molecular functions of KDM6A. Dual-luciferase reporter assay was used to determine the effects of KDM6A and FOXA1 on the promoters of the ARHGDIB and KDM6A.

**Results:**

We showed that the KDM6A inhibited the motility and invasiveness of the BCa cells. Mechanistically, KDM6A promotes the transcription of ARHGDIB by demethylating histone H3 lysine di/trimethylation (H3K27me2/3) and consequently leads to inhibition of Rac1. EZH2, which catalyses the methylation of H3K27, functions to silence ARHGDIB expression, and an EZH2 inhibitor can neutralize the metastatic effect caused by KDM6A deficiency. Furthermore, we demonstrated that FOXA1 directly binds to the KDM6A promoter and thus transactivates KDM6A, leading to diminished metastatic potential.

**Conclusion:**

Our findings establish the critical role of the FOXA1-KDM6A-ARHGDIB axis in restraining the malignancy of BCa and identify KDM6A and EZH2 as potential therapeutic targets in the management of BCa.

**Supplementary Information:**

The online version contains supplementary material available at 10.1186/s12943-021-01369-9.

## Background

Bladder cancer (BCa) has become the 10th most common malignancy, with high incidence and mortality worldwide. Approximately 75% of the patients diagnosed with BCa initially present with non-muscle invasive bladder cancer (NMIBC), and 25% present with muscle-invasive bladder cancer (MIBC) [1]. Among those patients with NMIBC (carcinoma in situ [CIS]/pTa/pT1), up to 30% of patients experience progression to MIBC, and the 5-year recurrence-free survival rate of MIBC is 74% for pT2, 52% for pT3, and 36% for pT4 [2]. Although approximately 50–60% of patients with MIBC can achieve an objective response, many patients remain resistant to first-line treatment [3]. With the rapid advancement of the comprehensive sequencing of genetic mutations and genomic expression of BCa, multiple commonly mutated genes such as tumour protein p53 (TP53), fibroblast growth factor receptor 3 (FGFR3), and phosphatidylinositol-4,5-bisphosphate 3-kinase catalytic subunit alpha (PIK3CA) have been identified and well studied [4]. However, there are still no reliable targets for the detection, treatment, or prognosis of BCa. Thus, a more detailed understanding of BCa pathogenesis and development is crucial for the clinical management of BCa.

Posttranslational modifications (PTMs) of histones are considered an important mechanism for the regulation of gene transcription [5]. Aberrant histone modifications have been widely implicated in the pathogenesis of several human diseases, and some of them have been proven to be potential diagnostic biomarkers or therapeutic targets. Lysine demethylase 6A (KDM6A), also known as the ubiquitously transcribed tetratricopeptide repeat on chromosome X (UTX), belongs to a family of JmjC domain-containing enzymes that mediate demethylation of H3K27me2/3 and therefore lead to transcriptional activation [6]. KDM6A plays an essential role during embryonic development [7]. De novo mutations of KDM6A are associated with Kabuki syndrome, a rare congenital anomaly syndrome characterized by intellectual disability, growth retardation, and multiple congenital abnormalities [8]. Somatic mutations in KDM6A have been found in a broad range of human cancers, including multiple myeloma, renal cell carcinoma, bladder cancer, head and neck squamous cell carcinoma, acute lymphoid leukaemia, prostate cancer, medulloblastoma, and pancreatic adenocarcinoma [9].

Although KDM6A mutations have been identified in a variety of cancers, the frequencies of the mutation are very different. KDM6A has the highest mutation frequency in BCa [7, 10]. Almost half of the NMIBC and a quarter of the MIBC cases were found to have KDM6A inactivating or deleterious mutations [4, 10]. Whether the KDM6A suppresses or promotes tumorigenesis and progression depends on the cancer type and its interacting transcription factors. For instance, KDM6A is a tumour suppressor in T-cell acute lymphoblastic leukaemia (T-ALL), except in T-ALL driven by TAL bHLH transcription factor 1 (TAL1) [11]. KDM6A has been shown to suppress the BCa cell proliferation and anchorage-independent growth in vitro, and urothelium-specific deletion of *kdm6a* increases BCa risk in female mice in vivo [12]. Moreover, the downregulation of KDM6A was shown to correlate tightly with the progression to advanced stages, and both the reduced mRNA levels and mutations of KDM6A predicted the poor outcome in BCa patients, suggesting the tumour-suppressive role of KDM6A in BCa [12, 13]. Despite the important roles of KDM6A in the tumorigenesis of BCa, its functions and detailed mechanisms in tumour progression and metastasis are poorly understood.

In this study, we found that KDM6A inhibited monolayer cell proliferation in a cell type-specific manner and decreased macrophage chemotaxis in BCa cells. Moreover, we showed that KDM6A inhibited migration and invasion of BCa cells in vitro and metastasis in vivo, and a low KDM6A expression level was correlated with the poor prognosis in patients with BCa. Importantly, we demonstrated that KDM6A exerted an antitumour effect by epigenetically activating Rho GDP dissociation inhibitor beta (ARHGDIB) transcription and consequently inhibiting Rac family small GTPase 1 (Rac1), which plays important roles in tumour cell motility, invasiveness, and metastasis. In addition, we found that forkhead box A1 (FOXA1) was recruited at the promoter of the KDM6A gene and promoted its transcription in BCa cells. Together, our findings establish a critical role of the FOXA1-KDM6A-ARHGDIB axis in the metastasis of BCa, thus suggesting a potential therapeutic implication in the management of BCa patients in the future.

## Methods

### Tissue specimens

This study was approved by the Medical Ethics Committee, Shandong University School of Clinical Medicine. A total of 12 human fresh BCa tissues were collected at Qilu Hospital of Shandong University. Informed consent was obtained from each patient before surgery. The tissue microarray, containing 46 BCa tissues and 10 normal bladder tissues, was obtained from Shanghai Outdo Biotech (Cat No. HBlaU066Su01, Shanghai, China).

### Cell cultures and reagents

Cell lines, such as T24, 5637, RT4, SV-HUC-1, HEK293T, HeLa, MKN-45, A549 and U2OS, were purchased from the American Type Culture Collect bioresource centre (Manassas, VA, USA). GSK126 (S7061)/SBE-β-CD (S4592) and MBQ-167 (S8749) were purchased from Selleck. T24, 5637, HeLa, MKN-45, and A549 cells were cultured in RPMI-1640 medium (Gibco, 11,875,093). The RT4 cells were cultured in McCoy’s 5A medium (Sigma Aldrich, M4892). The SV-HUC-1 cells were cultured in F12K medium (Macgene, CM10025). The HEK293T and U2OS cells were cultured in DMEM medium (Gibco, 11,995,065). All media were supplemented with 10% foetal bovine serum (Gibco, 10,099-141C). The cells were cultured at 37 °C in a humidified atmosphere with 5% CO_2_. The genomic characteristics of the BCa cell lines are presented in Table S1.

Stable KDM6A knockdown, overexpressed cell lines, and their controls were generated as described previously [14]. Briefly, pLVX-IRES-Puro-KDM6A, pMD2. G, pSPAX.2 plasmids were cotransfected into the HEK293T cells. Forty-eight hours later, the viral supernatant was collected, and the BCa cells were infected for 48 h. Then, the infected cells were cultured in the medium containing 2 mg/L puromycin (Solarbio, P8230) for 3 d. Adenoviruses encoding the GFP or KDM6A were purchased from Vigenebio (Jinan, China). The siRNA transfections were performed as previously described [15]. The siRNAs targeting FOXA1 and ARHGDIB were synthesized by RiboBio (Guangzhou, China). The target sequences were as follows (sense sequences): siFOXA1–1 (5′-GCACTGCAATACTCGCCTT-3′), siFOXA1–2 (5′-CCTCGGAGCAGCAGCATAA-3′), and siARHGDIB (5′-GGAAGGTTCTGAATATAGA-3′).

### Plasmids

The lentivirus vector-encoding human KDM6A (puro-KDM6A) was purchased from GeneChem Inc. (Shanghai, China). The KDM6A catalytic mutations (H1146A, E1148A) were introduced using a QuickMutation™ Site-Directed Mutagenesis Kit (Beyotime, China). The full-length FOXA1, ARHGDIB, upstream transcription factor 1 (USF1), upstream transcription factor 2 (USF2) and transcription factor binding to IGHM enhancer 3 (TFE3) complementary DNAs were amplified from T24 cells and cloned into the pcDNA3.1/Myc-His B vector (Invitrogen, V85520). The KpnI and XbaI fragment from puro-KDM6A was ligated into the pcDNA3.1/Myc-His B vector to generate pc3.1-KDM6A. The primers used for the construction of overexpression vectors are shown in Table S2.

The sequence of KDM6A shRNA (5′-AAGGAAATTCATTTACGACTT-3′) was synthesized by Sangon Biotech (Shanghai, China) and cloned into the lentiviral vector pLKO.1-Puro (Addgene, 8453). The enhancer of zeste 2 polycomb repressive complex 2 subunit (EZH2) knockdown lentivirus vectors were purchased from GeneChem Inc. (Shanghai, China). Different progressive deletions of the human KDM6A promoter fragments were amplified from T24 cells and cloned into the pGL3-Basic vector (Promega, E1751). The control plasmid pRL-TK was obtained from Promega (E2241).

### Nuclear and cytoplasmic protein extraction and Western blot

The nuclear and cytoplasmic proteins were obtained using the Nuclear and Cytoplasmic Protein Extraction Kit (Beyotime, P0028) following the manufacturer’s instructions. Western blotting was performed as described previously [14]. Briefly, equal amounts of extracts were loaded onto the SDS polyacrylamide gels, electrophoresed, and blotted onto the PVDF membranes (Millipore, IPVH00010). The membrane was blocked with 5% skimmed milk, followed by incubation with primary antibodies at 4 °C overnight. Then, the membranes were incubated with the HRP-conjugated secondary antibodies and detected using an ECL kit (Beyotime, P0018FM).

The primary antibodies included anti-KDM6A (CST, 33510), anti-ARHGDIB (Proteintech, 16,122–1-AP), anti-FOXA1 (Abcam, ab23738), anti-EZH2 (Abcam, ab228697), anti-H3K27me3 (Abcam, ab6002), anti-H3K27ac (CST, 8173), anti-H3K4me1 (CST, 5326), anti-Rac1 (Abcam, ab211161), anti-β-actin (Sigma Aldrich, A5441), anti-Histone H3 (Abways, CY6587) and anti-α-Tubulin (Santa Cruz, sc-32,293).

### RNA extraction, reverse transcription PCR, and ChIP assays

Extraction of the total RNA, reverse-transcription PCR, and real-time quantitative PCR (qPCR) were performed as described previously [14]. In brief, the total RNA was isolated using TRIzol reagent (Invitrogen, 15,596,026) according to the manufacturer’s instructions. One microgram of RNA was used to generate cDNA using the PrimeScript RT Reagent Kit (Accurate Biotechnology, AG11706) with random hexamers as primers. The qPCR was performed using the LightCycler 480 system (Roche, Mannheim, Germany). The qPCR primers used were purchased from Sangon Biotech, and the primer sequences are shown in Table S3.

Chromatin immunoprecipitation (ChIP) was performed as described previously [16]. Briefly, the cells were crosslinked with 1% formaldehyde. The DNA and proteins were broken down by ultrasonic shearing. The samples were centrifuged at 10000 g at 4 °C, and the supernatant was incubated with antibodies at 4 °C overnight. The complexes were washed with low- and high-salt buffers, and the DNA was extracted, precipitated, and detected by PCR. PCR primer sequences are provided in Table S4 and Table S5.

### MTT and colony formation assays

The 3-(4,5-Dimethylthiazol-2-yl)-2,5-diphenyl tetrazolium bromide (MTT) and colony formation assays were performed as previously described [15]. For the MTT assay, the MTT was added to each well at 37 °C for 4 h. The cells were washed with PBS once, 150 μL dimethyl sulfoxide (DMSO) was added, and the cells were shaken for 5 min. The optical density (OD) values were determined at 450 nm. For colony formation assay, 1000 cells per well were seeded in 6-well plates and cultured at 37 °C for 7–14 d. The cell colonies were stained with crystal violet and counted.

### Tumour xenograft models

Four-week-old female BALB/c (nu/nu) mice were purchased from Vital River Laboratory Animal Technology Co. Ltd. and raised under specific pathogen-free conditions. All experiments were approved by the Shandong University Animal Care Committee, and all procedures were performed in compliance with the institutional guidelines. The tumour xenograft model was generated as previously described [14]. In brief, for the subcutaneous tumour model, 1 × 10^6^ T24 cells were injected subcutaneously into the axillary fossa of each mouse. The volume of tumour nodules was measured every 4 days, and mice were sacrificed 28–32 d after implantation. The tumour nodules were excised, weighed, and embedded in paraffin for immunohistochemistry. The tumour volume was measured by a Vernier calliper and calculated by the following formula: V = (a × b^2^) / 2, where a and b represent the longest and shortest diameters, respectively.

For the tumour metastasis model, 1 × 10^6^ T24 cells were injected into the tail veins of nude mice, and the mice were sacrificed 4 weeks later. To determine whether GSK126 influences the lung metastasis of BCa, nude mice were administered with GSK126/20% SBE-β-CD by intraperitoneal injection at 50 mg/kg per day for 20 consecutive days. The mice were killed 5 weeks later, and the lungs were harvested.

### Immunohistochemistry (IHC)

Immunohistochemical staining was performed as described previously [14]. Briefly, IHC was performed using a PV-9001 kit (ZSGB-BIO) following the manufacturer’s instructions. The staining intensity was defined with a four-grade scoring system: 0 (negative), 1 (weak), 2 (moderate), and 3 (strong). The staining extent was quantified as five value grades: 0 (negative), 1 (1–25%), 2 (26–50%), 3 (51–75%), and 4 (76–100%). The sum of intensity and extent values was defined as the IHC score. The primary antibodies included anti-KDM6A antibody, anti-ARHGDIB antibody, and anti-Ki67 antibody (Invitrogen, PA5–19462).

### Isolation of murine bone marrow monocyte-derived macrophages (BMDMs)

BMDMs were obtained from C57BL6/J mice (8 weeks old). Mice were sacrificed, and bone marrow was flushed with PBS from tibiae and femurs. The cells were depleted of erythrocytes by the red blood cell lysis buffer treatment for 5 min and neutralized with PBS. Then, the cells were centrifuged at 1000 rpm and cultured in the RPMI-1640 medium supplemented with 10% foetal bovine serum and 15% L929 conditioned medium as a source of macrophage colony-stimulating factor [17]. The BMDMs were harvested after 7 days.

### Chemotaxis of macrophage assays

The BCa cells (5 × 10^4^) were seeded into the lower compartment of an 8.0-μm-pore Transwell system. The BMDMs (4 × 10^4^) were overlaid onto the upper chamber. After the incubation for 16 h, the macrophages were stained with crystal violet and counted.

### Enzyme-linked immunosorbent assays (ELISA)

The levels of interleukin-6 (IL-6) and C-C motif chemokine ligand 2 (CCL2) in the conditioned medium were determined using the ELISA reagent kits purchased from Elabscience (IL-6: E-EL-H0102c; CCL2: E-EL-H6005) following the manufacturer’s instructions. Briefly, the cell culture supernatants were collected and centrifuged at 1000 g for 20 min to eliminate cell debris. Then, 100 μL conditioned medium was added to the appropriate wells and incubated at 37 °C for 90 min. Then, the liquid was decanted, the biotinylated detection antibody working solution was added to the wells, and the mixture was incubated at 37 °C for 60 min. The wells were washed 3 times with wash buffer, the HRP conjugate working solution was added, and the wells were incubated at 37 °C for 30 min. Then, the wells were washed 5 times with wash buffer, and the substrate reagent was added for 15 min. The stop solution was added to the wells, and the OD values were determined at 450 nm.

### Wound healing and Transwell assays

Wound healing and Transwell assays were performed as previously described [14]. Briefly, for the wound healing assay, the BCa cells were plated in 24-well plates to form a full monolayer. A wound was created by scratching with a 200 μL pipette tip, and the intercellular distance was measured at 0 h and 24 h. The wound healing ratio was calculated as follows; migration distance / primary intercellular distance × 100%. For Transwell migration assay, 5 × 10^4^ cells in 200 μL serum-free medium were plated onto a Transwell chamber containing a polycarbonate membrane with 8.0 μm pores (BD Falcon, 353,097). The chamber was placed in a 24-well plate containing 600 μL medium with 10% foetal bovine serum at 37 °C and with 5% CO_2_. After the incubation period, non-migrated cells were detached using a cotton swab, and the cells adherent to the bottom of the membrane were fixed in 4% paraformaldehyde, stained with crystal violet, and counted. For the Transwell Matrigel assay, the polycarbonate membrane was covered with a layer of Matrigel matrix (Corning, 356,234), and then the following steps were the same as those in the Transwell migration assay.

### RNA-sequencing (RNA-seq) experiment and analysis

Total RNA was extracted from the KDM6A-overexpressing/knockdown and control T24 cells, and a cDNA library was prepared according to the standard Illumina RNA-seq instructions. Hisat2 was selected as the mapping tool because Hisat2 can generate a database of splice junctions based on the gene model annotation file. FeatureCounts v1.5.0-p3 was used to count the read numbers mapped to each gene. A fold change > 1.5 and false discovery rate (FDR) < 0.05 were set as the thresholds for identifying the differentially expressed genes (DEGs). Gene Ontology (GO) enrichment analysis and Kyoto Encyclopaedia of Genes and Genomes (KEGG) analysis of differentially expressed genes were performed using the ‘clusterProfiler’ R package.

### Dual-luciferase reporter assays

T24 cells were plated on 24-well plates at 1 × 10^5^ cells/well. Five-hundred nanograms of KDM6A promoter reporter plasmid and 50 ng of pRL-TK plasmid with or without 500 ng of transient expression plasmid were cotransfected per well using Lipofectamine 2000. After 48 h, the luciferase activities were determined using a dual-luciferase reporter assay kit (Promega, E1910) following the manufacturer’s instructions and measured by Centro XS LB 960 (Berthold Technologies). The firefly luciferase activity was normalized to Renilla luciferase for each well. The primers for the construction of KDM6A promoter vectors are shown in Table S6.

### Phalloidin staining

T24 cells were fixed in 3.7% methanol-free formaldehyde for 15 min, permeabilized in 0.1% Triton X-100 for 15 min, and stained with the fluorescent phalloidin solution (Invitrogen, A12379) for 1 h. Then, the T24 cells were washed 3 times with PBS and photographed under a fluorescence microscope (Olympus, Japan).

### Rac1 activation assays

The active form of Rac1 was isolated using a Rac1 activation assay kit (Abcam, ab211161) following the manufacturer’s instructions. Briefly, the cultured cells were suspended in 1× assay buffer on ice. The supernatants were collected after centrifugation at 13000 g for 10 min and incubated with the PAK1 PBD beads at 4 °C for 1 h with gentle agitation. Flowthroughs were washed 3 times with assay buffer and subjected to SDS-PAGE.

### Statistical analysis

All data were statistically analysed using SPSS v. 20.0 (IBM Corp, Armonk, NY, USA). The data are presented as the mean ± standard deviation (SD) or mean ± standard error of the mean (SEM). The data from the two groups were evaluated by a two-tailed unpaired Student’s t-test. The categorical data were analysed by the chi-square test. The correlation between the continuous variables was assessed by Spearman’s correlation analysis. The Kaplan-Meier survival curve was used to evaluate the survival rates in different groups, and the equivalences of the survival curves were tested by a log-rank test. *P* < 0.05 was considered statistically significant.

## Results

### KDM6A inhibits monolayer cell proliferation of BCa cells in a cell type-specific manner

**Fig. 1:**
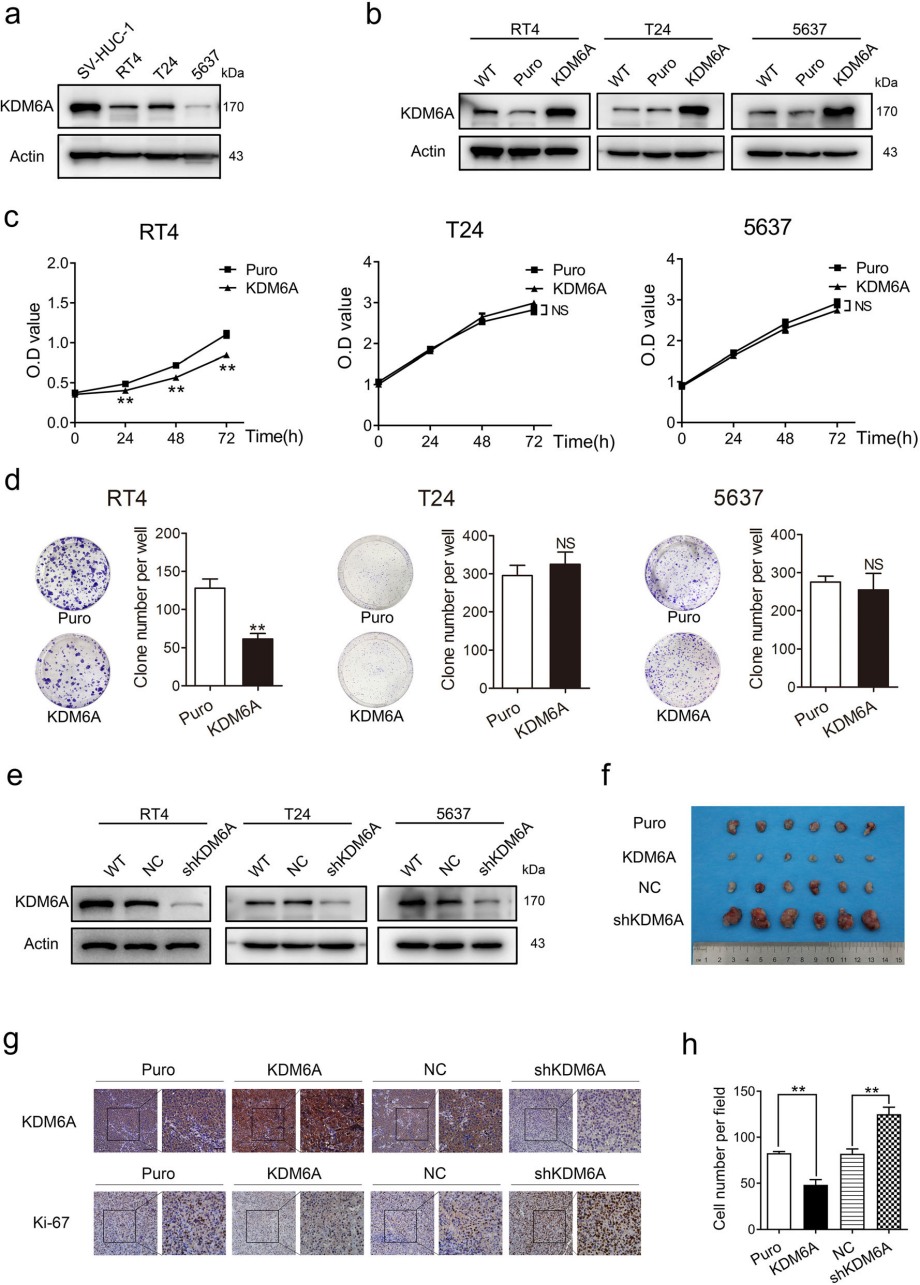
The role of KDM6A in proliferation of BCa cells. **a** The protein expression levels of KDM6A in the BCa cells were determined by Western blot. **b** The lentivirus mediated KDM6A overexpression in BCa cells were examined by Western blot. **c and d** Effect of KDM6A overexpression on cell proliferation was examined by MTT assays **(c)** and colony formation assays **(d)** in the BCa cells. **e** The protein expression levels of KDM6A in the indicated cells were determined by Western blot. **f** The image of subcutaneous tumours developed by the indicated T24 cells peeled from mice was shown. **g** The representative IHC images of KDM6A and Ki67 in subcutaneous tumours were shown. Scale bars, 50 μm (left) and 20 μm (right). **h** Cell migration assay of bone marrow monocyte derived-macrophages cocultured with conditioned medium (CM) from indicated cells. All quantification analyses were based on independent triplicate experiments. Error bars represent SD. **p < 0.01, NS no significant, based on Student’s t test

We first examined KDM6A expression in the immortalized human uroepithelial cell line SV-HUC-1 and 3 BCa cell lines, RT4 (NMIBC, wild-type KDM6A), 5637 (MIBC, wild-type KDM6A), and T24 (MIBC, mutant KDM6A). As shown in Fig. 1a and S1a, the protein and mRNA levels of KDM6A in SV-HUC-1 cells were much higher than those in the BCa cells. The T24 cells were reported to carry a homozygous KDM6A nonsense mutation (G2683T, NM_021140); however, Nickerson et al. demonstrated that a heterozygous KDM6A nonsense mutation existed in T24 cells [18, 19]. The results of Sanger sequencing showed that the T24 carried a heterozygous mutation of KDM6A at the reported locus, indicating that the T24 cells can express wild-type KDM6A (Fig. S1b). Next, we evaluated the role of KDM6A in BCa cell proliferation. The stable overexpressed KDM6A and control RT4, T24, and 5637 cells were established (Fig. 1b and S1c). The MTT and colony formation assays showed that the overexpression of KDM6A moderately inhibited the cell proliferation in RT4 cells; however, it did not affect the cell proliferation in T24 and 5637 cells (Fig. 1c and d). We further established KDM6A stable knockdown RT4, T24, and 5637 cells (Fig. 1e and S1d). Consistent with the results obtained in the overexpressed KDM6A cells, the knockdown of the KDM6A promoted the proliferation of RT4 cells but had no effect on the T24 and 5637 cells (Fig. S1e and f). To confirm the role of KDM6A in the proliferation of T24 and 5637 cells, we performed a transient adenovirus infection. Similar to the lentiviral transduction, KDM6A overexpression by adenovirus did not affect the cell proliferation in these cells (Fig. S1g-i). Together, these results indicate that the role of KDM6A in monolayer cell proliferation varies in different BCa cell lines.

Tumour xenograft models were employed to further evaluate the effect of the KDM6A on tumour growth in vivo. Although KDM6A did not affect the monolayer proliferation of T24 cells, the subcutaneous growth of the T24 cells in mice was significantly slower in overexpressed KDM6A cells but faster in the KDM6A knockdown cells (Fig. 1f and S1j-m). Accordingly, IHC staining showed dramatically increased Ki-67 expression in the KDM6A knockdown tumours but decreased Ki-67 expression in the overexpressed KDM6A tumours (Fig. 1g).

The interactions between the tumour cells and the microenvironment are key to tumour proliferation and progression in vivo, and the macrophages are one of the abundant cells in the tumour microenvironment (TME) [20]. Thus, we examined the role of KDM6A in modulating macrophage chemotaxis. Transwell assay showed that the overexpression of KDM6A markedly decreased, while the knockdown of KDM6A increased macrophage chemotaxis (Fig. 1h and S1n). F4/80, a macrophage-specific marker, was significantly increased in the KDM6A knockdown tumours, while KDM6A overexpression had no effect on it, which showed a low base level in control tumours (Fig. S1o). IL-6 and CCL2 were found to drive macrophage recruitment [20–22]. We then detected the levels of secreted IL-6 and CCL2 by ELISA. As shown in Fig. S1p and q, the concentrations of IL-6 and CCL2 were significantly decreased in the supernatant from the overexpressed KDM6A cells and increased in the supernatant from the KDM6A-knockdown cells. Furthermore, the qPCR analysis confirmed that the IL-6 and CCL2 mRNA levels were decreased in the overexpressed KDM6A cells but increased in the KDM6A-knockdown cells (Fig. S1r and s).

### KDM6A inhibits BCa cell migration and invasion in vitro and metastasis in vivo

**Fig. 2:**
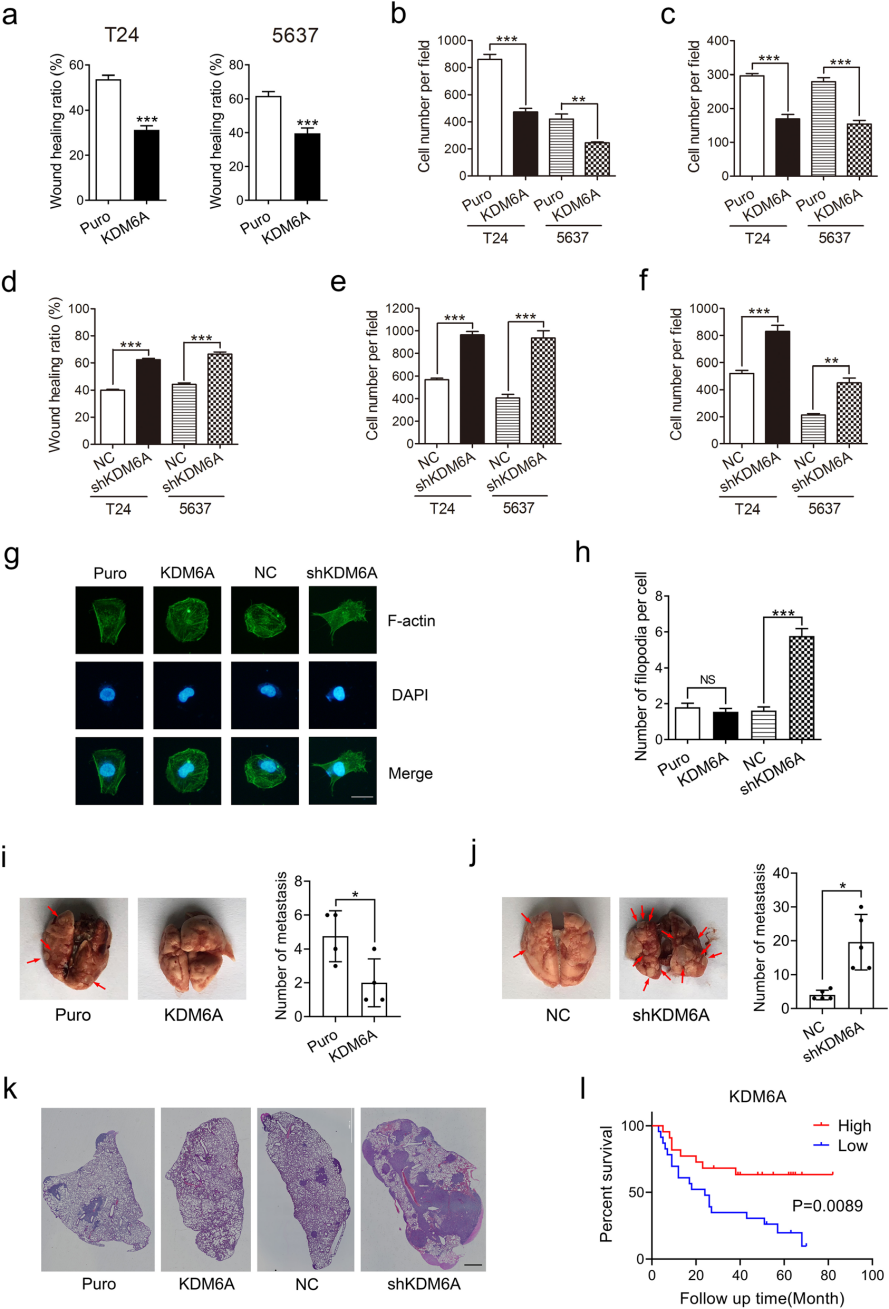
KDM6A inhibits BCa cell migration and invasion. **a-f** Migration and invasion capacities of indicated cells were examined by wound-healing assays **(a, d)**, Transwell migration assays **(b, e)**, and Transwell invasion assays **(c, f)**. The normalized wound area of control cells at 0 h was set as 1. **g** Representative image of filopodia staining in indicated cells were shown. Scale bars, 10 μm. **h** Quantification of the number of filopodia per cell was shown. All quantification analyses were based on independent triplicate experiments. Error bars represent SD. **i and j** Lung metastasis assays of indicated T24 cells. Representative gross lung images of indicated group were shown (left). The number of tumours per lung were counted (right, Puro vs KDM6A, *n* = 4, NC vs shKDM6A, *n* = 5). Error bars represent SEM. **k** Representative HE staining images of the lungs were shown. Scale bars, 200 μm. **l** Kaplan-Meier survival curve for overall survival of patients with BCa stratified by KDM6A expression levels from tissue microarray (Cat No. HBlaU066Su01; KDM6A-low, *n* = 23, KDM6A-high, *n* = 22). *p < 0.05, **P < 0.01, ***p < 0.001, NS no significant, based on Student’s t test. Kaplan-Meier analysis based on log-rank test

RT4, derived from grade-1 tumours, is a nonmetastatic BCa cell line [23]. Thus, we further examined the role of the KDM6A in BCa cell migration and invasion employing two MIBC cell lines, T24 and 5637. Wound healing assay showed that the stable overexpression of KDM6A significantly suppressed wound closure in both T24 and 5637 cells (Fig. 2a and S2a). Transwell and Matrigel invasion assays demonstrated that the stable overexpression of KDM6A significantly reduced the cell migration and invasion ability (Fig. 2b-c and S2b-c). Consistent with these results, transient upregulation of KDM6A by adenovirus also dramatically inhibited cell migration and invasion in both T24 and 5637 cells (Fig. S2d-f). In contrast, the knockdown of KDM6A promoted cell migration and invasion in both BCa cell lines (Fig. 2d-f and S2g-i). Together, these results indicate that KDM6A inhibits BCa cell migration and invasion in vitro.

Epithelial-mesenchymal transition (EMT) and matrix metalloproteinases (MMPs) are highly associated with tumour cell migration, invasion, and metastasis. No significant changes in the mRNA levels of the EMT markers were observed in the overexpressed KDM6A and KDM6A-knockdown BCa cells (Fig. S2j and k). Although the levels of MMP1, MMP2, and MMP9 were downregulated in the KDM6A-knockdown T24 cells, they were unaffected by KDM6A overexpression (Fig. S2l). These data suggest that KDM6A inhibits the migration and invasion of BCa cells in an EMT- and MMP-independent manner. Platinum-based combination chemotherapy has been used as the first choice for preventing tumour relapse in patients with metastatic disease [2]. Our data showed that both overexpression and knockdown of KDM6A had no effect on cisplatin sensitivity in T24 cells (Fig. S2m and n).

Cell migration could be mediated by actin-myosin cytoskeletal regulation, and an increasing number of filopodia that protrude from the cell surface has been shown to be functionally crucial for tumour invasiveness [24]. Although the overexpression of KDM6A had no influence on filopodia formation in T24 cells, which showed a low basal number of filopodia, the knockdown of KDM6A markedly increased the formation of filopodia (Fig. 2g and h).

To confirm the role of KDM6A in tumour metastasis in vivo, we performed tail vein xenografts in BALB/c (nu/nu) mice. The overexpressed KDM6A T24 cells developed approximately 55% fewer lung metastasis foci than the control cells (Fig. 2i and k). In contrast, KDM6A knockdown resulted in a significant increase in lung metastases (Fig. 2j and k). To verify the role of KDM6A in predicting clinical outcome in BCa patients, we performed IHC on a tissue microarray containing 56 human bladder cancer samples and found that low KDM6A protein expression was associated with poorer overall survival (OS) (Fig. 2l and S2o). Furthermore, the Human Protein Atlas website (https://www.proteinatlas.org/) was employed to determine the prognostic value of KDM6A expression based on The Cancer Genome Atlas (TCGA) database. The results also confirmed that the low KDM6A mRNA levels could reflect the poorer prognosis of BCa patients (Fig. S2p).

### Identification of ARHGDIB as a target of KDM6A in BCa cells

**Fig. 3:**
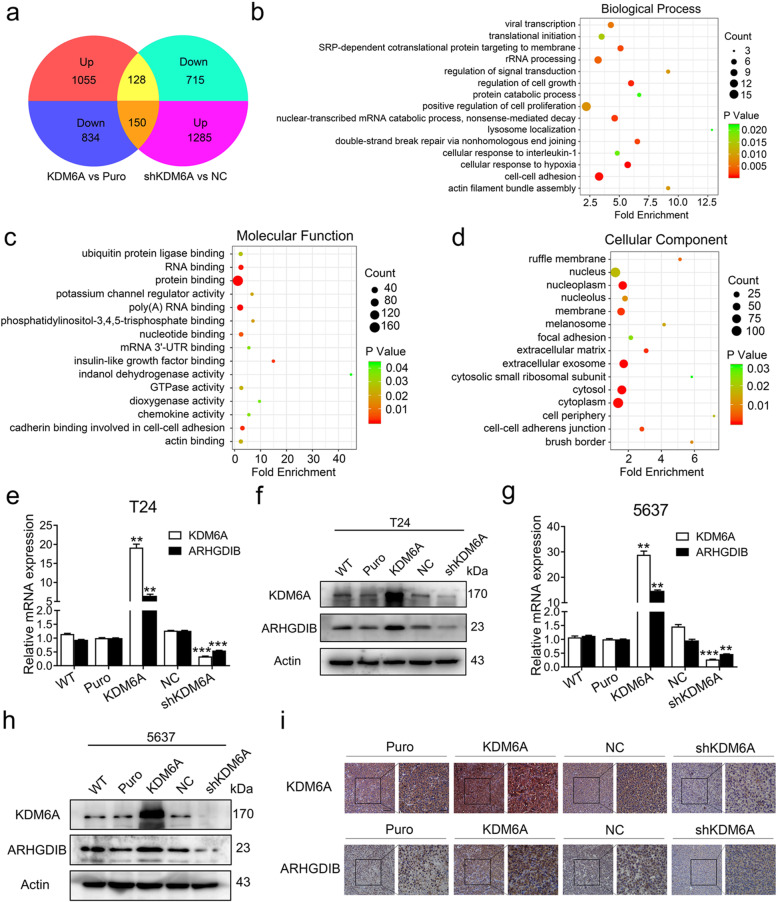
KDM6A promotes ARHGDIB transcription in BCa cells. **a** A Venn diagram comparing depicted 278 genes as potential direct targets of KDM6A in T24 cells based on RNA-seq data. **b-d** Gene Ontology (GO) analysis of the 278 genes. Dot plots of top 15 most significantly affected categories were shown. Biological Process **(b)**, Molecular Function **(c)** and Cellular Component **(d)**. **e-h** KDM6A and ARHGDIB expression levels in indicated cells. mRNA levels were detected by qPCR (**e, g**, Puro was set as 1, KDM6A vs Puro, shKDM6A vs NC), and protein levels were determined by Western blot **(f, h)**. **i** Representative IHC images of KDM6A and ARHGDIB in subcutaneous tumours were shown. Scale bars, 50 μm (left) and 20 μm (right). All quantification analyses were based on independent triplicate experiments. Error bars represent SD. **p < 0.01, ***p < 0.001, NS no significant, based on Student’s t test

To elucidate the mechanisms underlying the regulation of KDM6A on cell migration and invasion in BCa, we performed RNA-seq to assay the transcriptomes of overexpressed KDM6A and KDM6A-knockdown T24 cells. The overexpression of KDM6A led to 1055 upregulated genes and 834 downregulated genes, while the knockdown of KDM6A resulted in 1285 upregulated genes and 715 downregulated genes (Fig. 3a and S3a, Appendix S1 and S2). qPCR was performed to confirm the RNA-seq results (Fig. S3b and c).

A Venn diagram was constructed based on the RNA-seq results, and 278 DEGs were identified (Fig. 3a). GO analysis was carried out to investigate the biological processes and molecular functions in which these DEGs might participate (Fig. 3b-d). The GO terms “viral transcription”, “translational initiation”, and “SRP-dependent cotranslational protein targeting to membrane” were most enriched in biological processes, “ubiquitin-protein ligase binding”, “RNA binding”, and “protein binding” were the top three enriched terms in molecular functions, and “ruffle membrane”, “nucleus”, and “nucleoplasm” were frequently enriched in the cellular components. Importantly, some significantly enriched GO terms were closely related to cell migration and invasion, such as “cell-cell adhesion”, “actin filament bundle assembly”, “focal adhesion”, “cell-cell adherens junction”, “cadherin binding involved in cell-cell adhesion”, and “actin-binding”.

ARHGDIB, also known as RhoGDI2, is a guanosine diphosphate (GDP) dissociation inhibitor (GDI) that has been reported to act as a functional metastasis suppressor and prognostic marker in human BCa [25, 26]. RNA-seq analysis showed that ARHGDIB was one of the top 10 DEGs; thus, we examined the effect of KDM6A on ARHGDIB expression by qPCR and Western blot. The results revealed that the overexpression of KDM6A significantly increased and the knockdown of KDM6A decreased both the mRNA and protein levels of ARHGDIB in T24 and 5637 cells (Fig. 3e-h). The KDM6A-ARHGDIB axis was further confirmed by IHC in the tumour xenograft models (Fig. 3i). ARHGDIB belongs to the RhoGDI family, including Rho GDP dissociation inhibitor alpha (ARHGDIA), ARHGDIB, and Rho GDP dissociation inhibitor gamma (ARHGDIG). However, the overexpression or knockdown of KDM6A did not affect the ARHGDIA and ARHGDIG mRNA levels, suggesting the specific role of KDM6A in ARHGDIB regulation in BCa cells (Fig. S3d and e). To identify whether the KDM6A-ARHGDIB axis is a general phenomenon, KDM6A was overexpressed in HeLa (cervical cancer cell line), MKN-45 (gastric cancer cell line), and A549 (non-small-cell lung cancer cell line) cells. As shown in Fig. S3f, the overexpression of KDM6A only slightly increased the ARHGDIB levels in MKN-45 cells, indicating that KDM6A regulates ARHGDIB in a cancer type-specific manner.

### ARHGDIB acts as a downstream effector of KDM6A to mediate the inhibition of cell migration and invasion

**Fig. 4:**
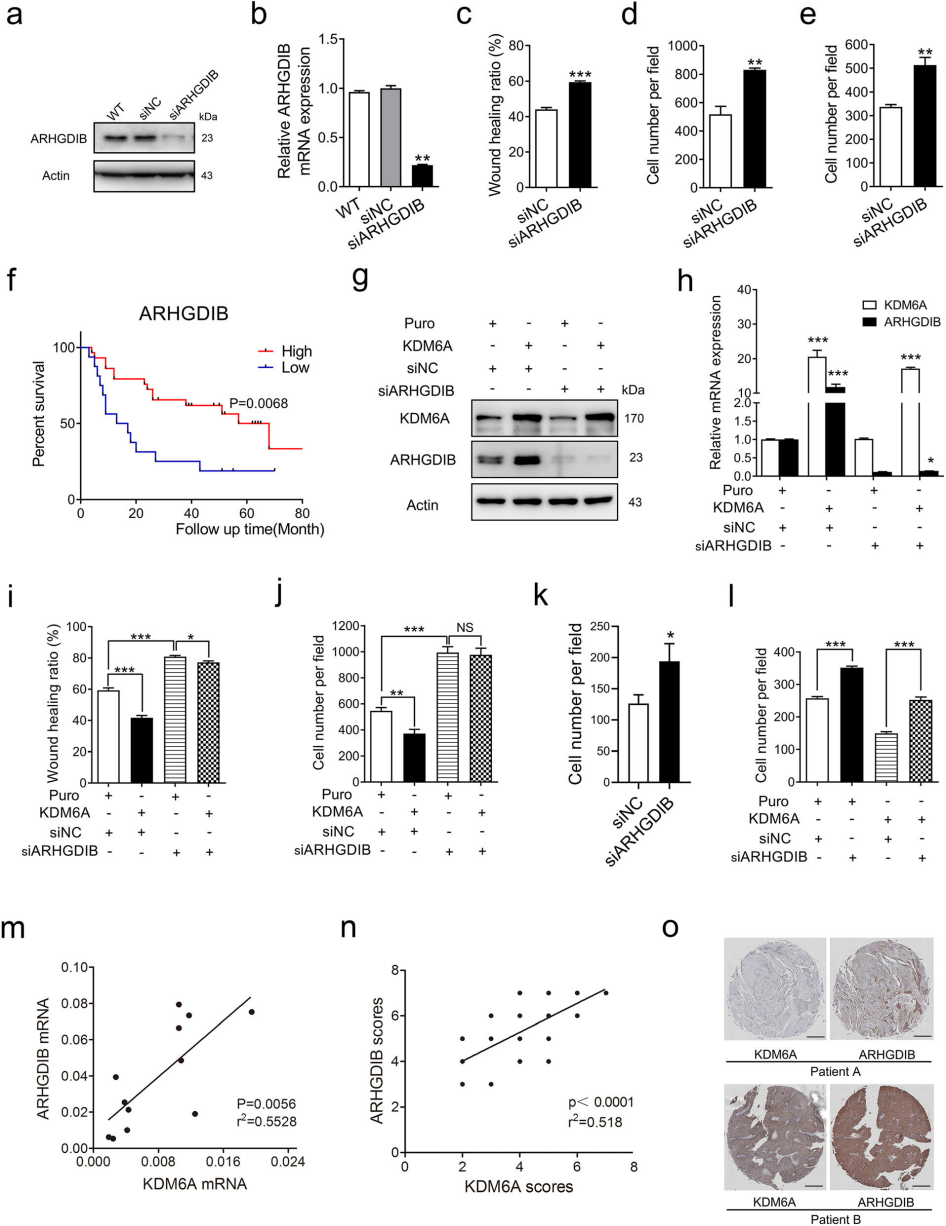
ARHGDIB acts as a downstream effector of KDM6A to mediate the inhibition of cell migration and invasion. **a and b** siRNA mediated ARHGDIB knockdown in T24 cells was verified by Western blot **(a)** and qPCR **(b)**, siNC was set as 1. **c-e** Migration and invasion capacities of the indicated cells were determined by wound-healing assays **(c)**, Transwell migration assays **(d)**, and Transwell invasion assays **(e)**. **f** Kaplan-Meier survival curve for overall survival of the patients with BCa stratified by ARHGDIB expression levels from tissue microarray (Cat No. HBlaU066Su01; ARHGDIB-low, *n* = 16, ARHGDIB-high, *n* = 29). **g** The protein levels of KDM6A and ARHGDIB in indicated cells were assessed by Western blot. **h** The mRNA levels of KDM6A and ARHGDIB in indicated cells were detected by qPCR, Puro + siNC was set as 1, Puro + siNC vs KDM6A + siNC, Puro + siARHGDIB vs KDM6A + siARHGDIB. **i and j** Wound healing assays **(i)** and Transwell invasion assays **(j)** of the effects of ARHGDIB knockdown on cell migration and invasion. **k and l** Cell migration assay of bone marrow monocyte-derived macrophages cocultured with conditioned medium (CM) from indicated cells. **m** The correlation between KDM6A and ARHGDIB mRNA levels in 12 human fresh BCa tissues was assessed by Spearman’s correlation analysis. **n** The correlation between KDM6A and ARHGDIB protein levels in tissue microarray (Cat No. HBlaU066Su01) was assessed. **o** Representative IHC images of KDM6A and ARHGDIB in tissue microarray were shown, Scale bar, 500 μm. All quantification analyses were based on independent triplicate experiments. Error bars represent SD. *p < 0.05, **p < 0.01, ***p < 0.001, NS no significant, based on Student’s t test. Kaplan-Meier analysis based on log-rank test

To evaluate the role of ARHGDIB in the biological function of KDM6A in BCa cells, ARHGDIB was knocked down and verified by Western blot and qPCR in T24 cells (Fig. 4a and b). Wound-healing, Transwell and Matrigel invasion assays revealed that the downregulation of ARHGDIB by siRNA produced similar changes in migration and invasion ability to that of KDM6A knockdown (Fig. 4c-e and S4a-c). The IHC analysis of the tissue microarray showed that lower ARHGDIB protein levels reflected the poorer prognosis of BCa patients (Fig. 4f). Moreover, the ARHGDIB mRNA levels were also significantly associated with the OS in BCa patients based on an analysis of the TCGA database (Fig. S4d). Importantly, the knockdown of ARHGDIB abrogated the decreased migration and invasion induced by the overexpression of KDM6A in T24 cells (Fig. 4g-j and S4e-f). Furthermore, we investigated the role of ARHGDIB in macrophage chemotaxis. The knockdown of ARHGDIB in T24 cells displayed a similar trend for macrophage chemotaxis as KDM6A knockdown (Fig. 4k and S4g). Importantly, the knockdown of ARHGDIB reduced macrophage chemotaxis caused by the overexpression of KDM6A (Fig. 4l and S4h), indicating that KDM6A-dependent macrophage chemotaxis regulation is, at least partly, mediated by regulating ARHGDIB expression.

To further confirm the correlation in human BCa, we examined the KDM6A and ARHGDIB mRNAs in 12 human BCa samples. The results showed that the mRNA levels of KDM6A and ARHGDIB were positively correlated (Fig. 4m). Moreover, the IHC analysis of the tissue microarray indicated that the protein levels of KDM6A and ARHGDIB were highly consistent in the BCa tissues (Fig. 4n and o). The relationships between the clinicopathological parameters and KDM6A or ARHGDIB were further tested using the TCGA database. As shown in Fig. S4i-l, the BCa clinical stage was significantly related to both KDM6A and ARHGDIB expression levels. Together, these results demonstrate that KDM6A inhibits cell migration and invasion by enhancing ARHGDIB expression in BCa cells.

### Metastasis-suppressive effect of KDM6A associates with the inhibition of Rac1

**Fig. 5:**
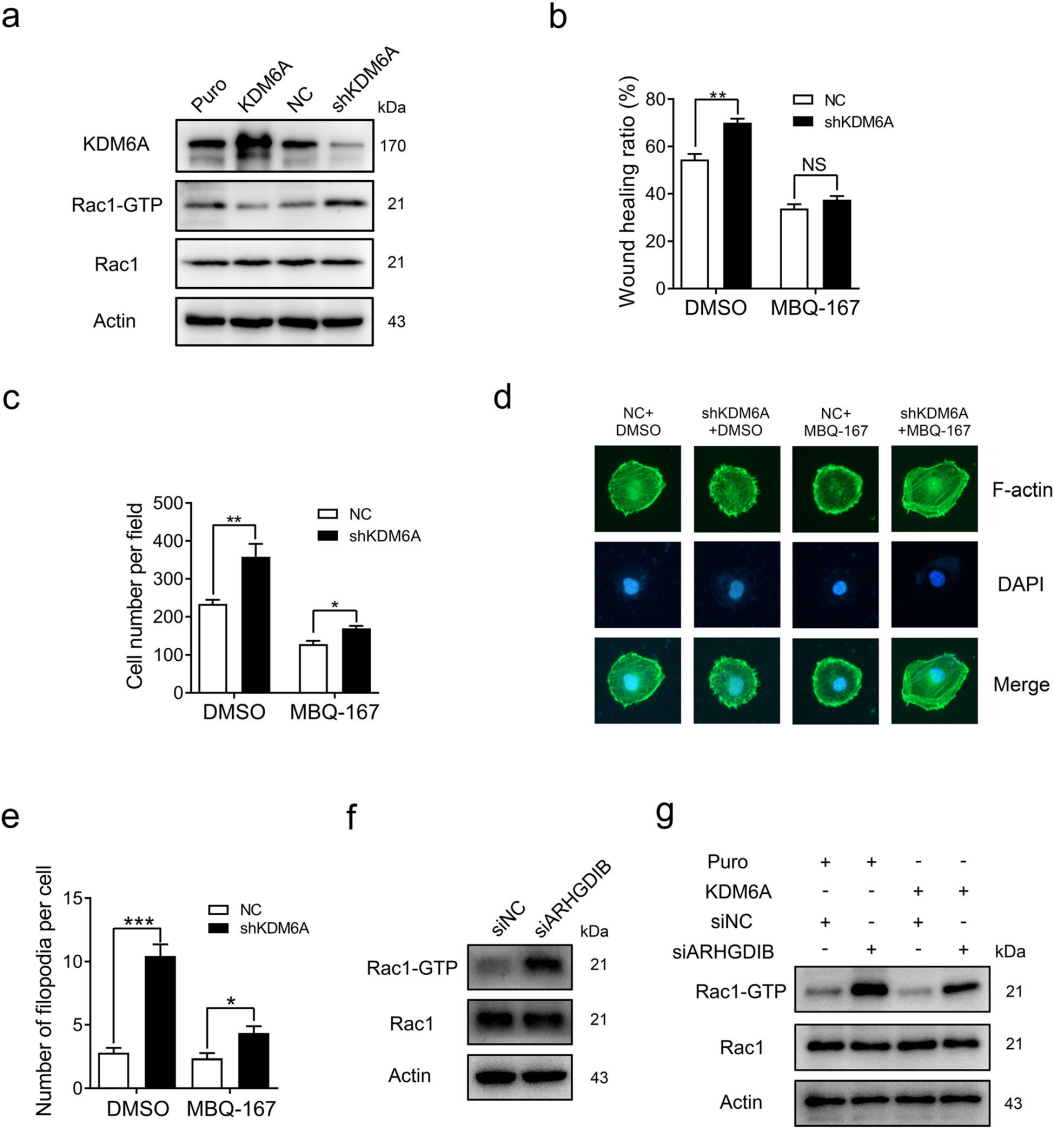
KDM6A suppresses BCa cell migration and invasion by inhibiting Rac1. **a** The protein levels of total and active Rac1 (Rac1-GTP) in KDM6A overexpression and knockdown T24 cells were determined by Western blot. **b and c** The cell motility of indicated T24 cells treated with MBQ-167 (200 nM) or DMSO for 48 h was evaluated by wound healing assays **(b)** and Transwell invasion assays **(c)**. **d** Representative image of filopodia staining in indicated cells were shown. Scale bars, 10 μm. **e** Quantification of the number of filopodia per cell was shown. **f** The protein levels of total and active Rac1 (Rac1-GTP) in indicated T24 cells were measure by Western blot. **g** The levels of total and active Rac1 (Rac1-GTP) in indicated cells were determined by Western blot. All quantification analyses were based on independent triplicate experiments. Error bars represent SD. *p < 0.05, **p < 0.01, ***p < 0.001, NS no significant, based on Student’s t test

ARHGDIB has been identified as a critical inhibitor of Rho GTPases, through which ARHGIDB suppresses tumour metastasis [27]. Given that KDM6A promotes ARHGDIB transcription and the DEGs associated with KDM6A in the RNA-seq analysis were significantly enriched in “GTPase activity” based on GO analysis (Fig. 3c), we next determined whether KDM6A modulated the activity of Rac1, a major binding substrate of ARHGDIB, which is a crucial component of tumorigenesis and metastasis [28, 29]. Although the total Rac1 protein levels did not change, the Rac-GTP pulldown assay showed that the upregulation of KDM6A significantly decreased the amount of active Rac1, while the knockdown of KDM6A resulted in the opposite effect (Fig. 5a).

To determine whether the metastasis-suppressive effect of KDM6A depends on Rac1 suppression, a Rac1 inhibitor, MBQ-167, was employed. As shown in Fig. S5a, although 200 nM MBQ-167 treatment did not robustly reduce cell proliferation in T24 cells, MBQ-167, at least in part, limited the increased migration, invasion, and filopodia formation caused by KDM6A knockdown (Fig. 5b-e and S5b-c), suggesting that the ability of KDM6A to suppress metastasis is attributable, in significant part, to its capacity to inhibit Rac1.

We further verified the role of ARHGDIB in Rac1 inhibition by KDM6A. As expected, the knockdown of ARHGDIB increased Rac1 activity (Fig. 5f). Importantly, ARHGDIB knockdown limit the decrease in active Rac1 in KDM6A-overexpressed cells, suggesting that the KDM6A-dependent upregulation of ARHGDIB is associated with effective inhibition of active Rac1 (Fig. 5g). A previous study demonstrated that KDM6A promoted a dedicator of cytokinesis (DOCK) 5/8 transcription and Rac GTPase activation to promote tumour metastasis in acute myeloid leukaemia [30]. The qPCR results showed that KDM6A did not affect the DOCK5 and DOCK8 levels in T24 cells, indicating that KDM6A regulates Rac1 activity in a cancer type-dependent manner (Fig. S5d-e). Taken together, our data demonstrate that the ARHGDIB-Rac1 axis contributes to metastasis suppression by KDM6A in BCa cells.

### KDM6A promotes ARHGDIB expression in a histone demethylase-dependent manner

**Fig. 6:**
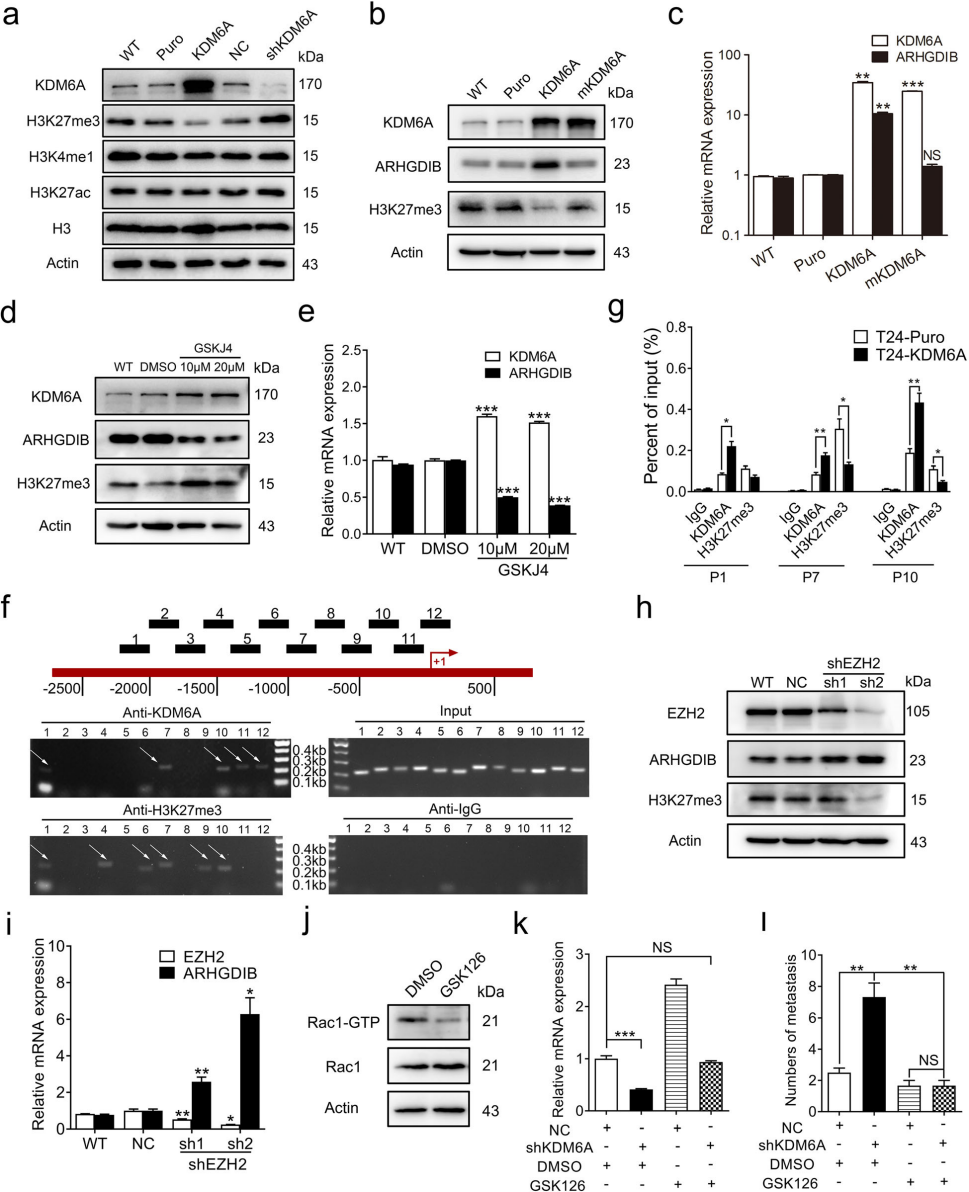
KDM6A promotes ARHGDIB transcription by catalyzing demethylation of H3K27me3. **a** The indicated protein levels in Puro/KDM6A and NC/shKDM6A T24 cells were detected by Western blot. **b** and **c** Western blot (**b**) and qPCR (**c**, Puro was set as 1) were performed to detect indicated gene levels in KDM6A catalytic domain wild type or mutant expressed T24 cells. **d** and **e** The effect of GSKJ4 on ARHGDIB expression levels in T24 cells was evaluated by Western blot **(d)** and qPCR (**e**, DMSO was set as 1). **f** Schematic diagram showed the location of 12 pairs of primers in ARHGDIB promoter regions (up). ChIP assays were performed using KDM6A and H3K27me3 antibody in T24 cells to detect the binding sites in ARHGDIB promoter regions (down). **g** ChIP-qPCR assays were performed using KDM6A and H3K27me3 antibody in indicated T24 cells. The normalized expression in Puro/KDM6A cells (input) was set as 1, respectively. **h** and **i** The levels of ARHGDIB in EZH2 knockdown T24 cells were detected by Western blot **(h)** and qPCR (**i**, NC was set as 1). **j** The protein levels of total and active Rac1 in T24 cells treated with GSK126 (20 μM) for 48 h were determined by Western blot. **k** The mRNA levels of ARHGDIB in indicated T24 cells treated with GSK126 (20 μM) for 48 h were measured by qPCR (NC + Control was set as 1). All quantification analyses were based on independent triplicate experiments. Error bars represent SD. **l** Lung metastasis assays of indicated cells. The number of lung metastasis node were counted (Puro, n = 4, KDM6A, *n* = 3, NC, n = 3, shKDM6A, n = 3). Error bars represent SEM. *p < 0.05, **p < 0.01, ***p < 0.001, NS no significant, based on Student’s t test

KDM6A, a histone demethylase, activates gene transcription by antagonizing EZH2 through the removal of methyl groups from H3K27 [31, 32]. Loss of KDM6A amplifies polycomb repressive complex 2 (PRC2)-regulated transcriptional repression in BCa [32]. KDM6A has also been shown to be involved in the establishment of the active histone marks H3K4 methylation and H3K27 acetylation in a demethylase activity-independent manner via recruitment of the COMPASS-like complex and histone acetyltransferase p300 [10, 33]. We further investigated the mechanisms by which ARHGDIB expression is regulated by KDM6A in BCa cells. First, we clarified the subcellular location of KDM6A by Western blot. Both endogenous and exogenous KDM6A were mainly located in the nucleus (Fig. S6a). The overexpression of KDM6A decreased and the knockdown of KDM6A increased global H3K27me3 levels in both T24 and 5637 cells (Fig. 6a and S6b). However, the global levels of monomethylated H3K4 (H3K4me1) and acetylated H3K27 (H3K27ac) were not changed by KDM6A (Fig. 6a and S6b). Additionally, the levels of EZH2 were not changed (Fig. S6c), indicating that the regulation of H3K27me3 levels by KDM6A was not due to a change in EZH2 expression. A demethylase inactive mutant, mKDM6A, was then expressed. In contrast to wild-type KDM6A, mKDM6A lost its histone demethylase activity and failed to upregulate ARHGDIB expression at both the protein and mRNA levels (Fig. 6b and c). GSKJ4, an H3K27 demethylase inhibitor of KDM6A, significantly repressed ARHGDIB expression in T24, 5637 and SV-HUC-1 cells (Fig. 6d-e and S6d-e). Similar to the observation that KDM6A regulated ARHGDIB expression in a cancer-specific manner (Fig. S3e), among all four cell lines for different cancer types, the GSKJ4 treatment only inhibited ARHGDIB expression in HeLa and MKN-45 cells (Fig. S6f-i). Together, these results suggest that the regulation of ARHGDIB expression by KDM6A depends on its demethylase catalytic activity.

Next, we performed a ChIP assay and found that both KDM6A and H3K27me3 signals were observed in the ARHGDIB promoter region (Fig. 6f). Importantly, the enrichment of KDM6A was increased, while the enrichment of H3K27me3 was significantly decreased in the ARHGDIB promoter region in KDM6A-overexpressed T24 cells (Fig. 6g), indicating that KDM6A promotes ARHGDIB transcription by directly binding to and demethylating H3K27 at the ARHGDIB promoter.

We next determined whether EZH2 was also involved in ARHGDIB regulation. Both the protein and mRNA levels of ARHGDIB were upregulated in EZH2 knockdown T24 cells (Fig. 6h and i). Moreover, treatment with the EZH2 inhibitor GSK126 significantly reduced Rac1 activity (Fig. 6j). Importantly, GSK126 completely blocked the decrease in ARHGDIB mRNA levels caused by KDM6A knockdown in T24 cells (Fig. 6k).

To confirm the role of EZH2 in KDM6A-mediated BCa metastasis inhibition, GSK126 was administered by intraperitoneal injection after T24 cells were injected into the tail vein of mice. The knockdown of KDM6A significantly increased the amount of metastasis in the lungs, while GSK126 markedly reversed these effects (Fig. 6l and S6j). Insulin-like growth factor binding protein 3 (IGFBP3), which is associated with BCa development, was reported to be regulated by EZH2 and contributed to EZH2 inhibition-mediated antiproliferative activity in KDM6A-null BCa cells; additionally, the depletion of KDM6A increased the binding of H3K27me3 at the IGFBP3 gene promoter in RT4 cells [32]. However, although another EZH2 target gene, cyclin-dependent kinase inhibitor 2A (CDKN2A), was positively regulated by KDM6A, no changes in IGFBP3 mRNA levels were observed in the KDM6A-overexpressed or KDM6A-knockdown T24 cells (Fig. S6k and l). Together, these results indicate that KDM6A acts antagonistically to EZH2 in regulating ARHGDIB transcription to suppress the metastasis of BCa cells.

### FOXA1 binds to the KDM6A promoter and promotes its transcription

**Fig. 7:**
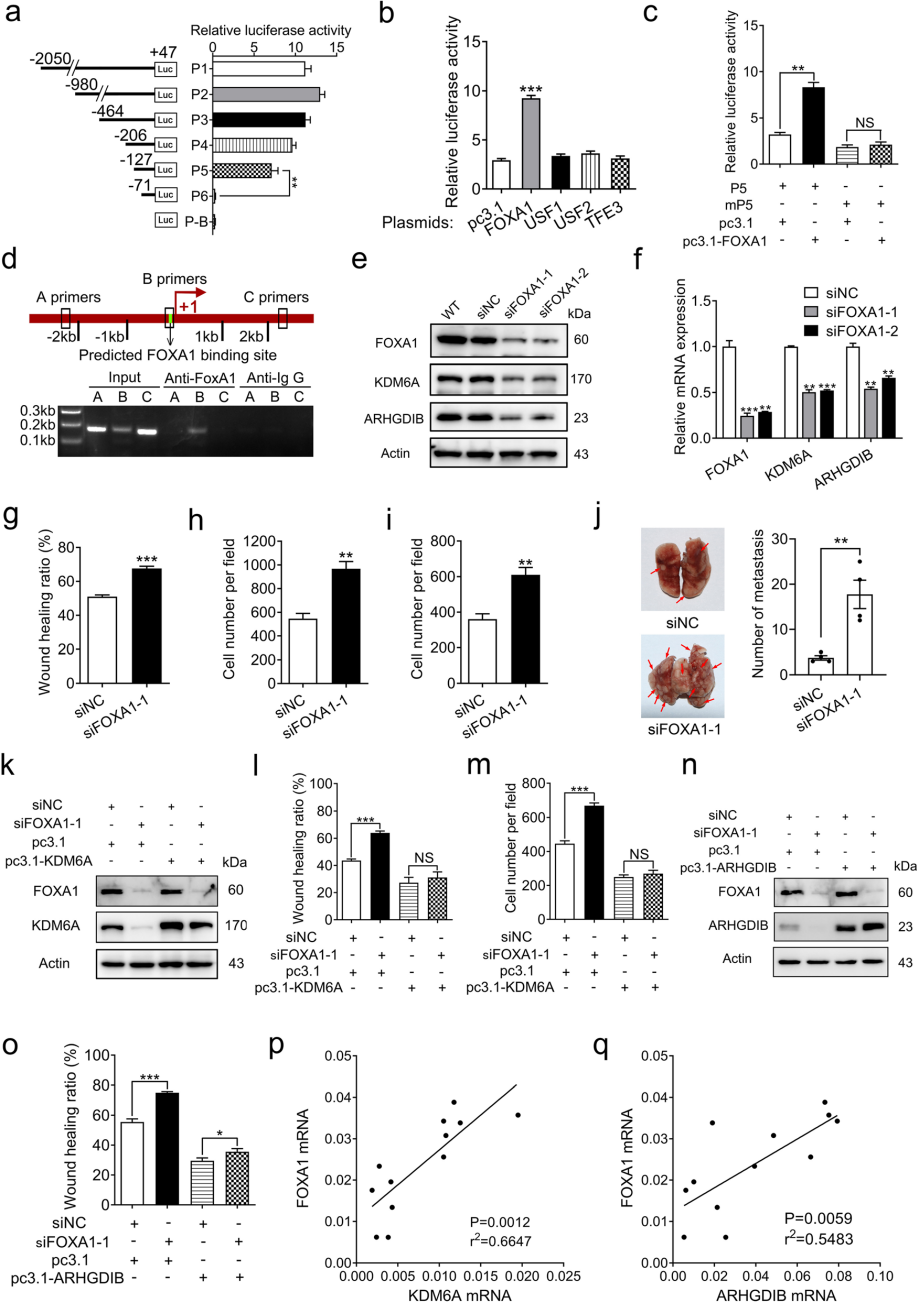
FOXA1 binds to the KDM6A promoter and active KDM6A transcription. **a** Schematic diagram of KDM6A promoter fragments cloned into pGL3-basic vector (left). Transcriptional activities of KDM6A promoter fragments were examined by dual-luciferase reporter assays in T24 cells (right). **b** The relative luciferase activities of P5 reporter in T24 cells transiently transfected with indicated plasmids were examined by dual-luciferase reporter assays. **c** The relative luciferase activities of wild type (P5) and FOXA1-binding site mutant (mP5) KDM6A promoter fragments containing reporters in T24 cells transiently transfected with pc3.1 of pc3.1-FOXA1 were examined by dual-luciferase reporter assays. **d** Schematic diagram showed the locations of target primer (B primer) and two pairs of negative control primers (A and C primers) on KDM6A promoter region (up). ChIP assays was performed using FOXA1 antibody in wild type T24 cells (down). **e** and **f** KDM6A and ARHGDIB expression in indicated T24 cells were detected by Western blot (**e**) and qPCR (**f**, siNC was set as 1). **g-i** Migration and invasion capacities of indicated cells were examined by wound-healing assays (**g**), Transwell migration assays (**h**) and Transwell invasion assays **(i)**. The normalized wound area of control cells at 0 h was set as 1. **j** Lung metastasis assays of indicated T24 cells. Representative gross lung images of indicated group were shown (left). The number of tumours per lung were counted (right, siNC vs siFOXA1–1, n = 4). Error bars represent SEM. **k** The FOXA1 and KDM6A protein levels in indicated T24 cells were detected by Western blot. **l** and **m** The migration and invasion capacities of indicated cells were examined by wound healing assays (**l**) and Transwell invasion assays (**m**). **n** The FOXA1 and ARHGDIB protein levels in indicated T24 cells were detected by Western blot. **o** The migration capacities of indicated cells were examined by wound healing assays. **p** and **q** The corelation between FOXA1 and KDM6A (**p**), FOXA1 and ARHGDIB (**q**) mRNA levels in 12 fresh human BCa tissues was assessed by Spearman’s correlation analysis. All quantification analyses were based on independent triplicate experiments. Error bars represent SD. *p < 0.05, **p < 0.01, ***p < 0.001, NS no significant, based on Student’s t test

Although KDM6A plays an important role in tumour progression, the regulatory mechanism of KDM6A is poorly understood. We then determined the mechanism governing KDM6A transcription in BCa cells. Luciferase reporters containing different progressive deletions of human KDM6A promoter fragments between − 2050 and + 47 bp upstream of the KDM6A transcriptional start site were constructed (Fig. 7a). Dual-luciferase reporter assay showed that no significant change in reporter activity was detected among constructs P1-P5 (Fig. 7a). However, the luciferase activities profoundly decreased from P5 to P6 (Fig. 7a), which showed only a background level of luciferase activity, indicating that the fragment between − 127 and − 71 bp contains cis elements that are essential for driving the transcription of KDM6A.

Inspecting the sequences of this region using the JASPAR database identified the FOXA1, USF1, USF2, and TFE3 binding sites within this region (Fig. S7a). We then transiently cotransfected the FOXA1-, USF1-, USF2- or TFE3-expressing plasmids with the P5 reporter into T24 cells and found that the luciferase activity was upregulated only by FOXA1 overexpression (Fig. 7b and S7b). To confirm that this putative FOXA1-binding site mediates the transcriptional activity of the KDM6A promoter, point mutations were introduced, and a P5 mutant construct (mP5) was generated (Fig. S7c). As shown in Fig. 7c, the mutation of the FOXA1 binding site blocked the increase in the luciferase activity induced by FOXA1 overexpression. Moreover, a ChIP assay showed that FOXA1 could bind to the promoter of KDM6A (Fig. 7d). We then performed knockdown for the FOXA1 with siRNAs. As expected, downregulating FOXA1 significantly decreased KDM6A and consequently reduced the ARHGDIB expression levels in both T24 and 5637 cells (Fig. 7e-f and S7d-e). Collectively, these results suggest that FOXA1 binds directly to the promoter of KDM6A and activates KDM6A transcription.

Loss of FOXA1 is associated with high grade, late-stage prognosis in patients with BCa [34, 35]. However, the mechanisms and targets of FOXA1 in BCa are still poorly understood. The finding that FOXA1 activates KDM6A transcription prompted us to investigate whether the KDM6A-ARHGDIB axis mediates the tumour-suppressive effect of FOXA1 in BCa cells. The knockdown of FOXA1 in T24 cells significantly increased cell migration and invasion in vitro and markedly promoted the lung metastasis potential in vivo (Fig. 7g-j and S7f-h). These results are quite similar to those for KDM6A knockdown. Importantly, the overexpression of KDM6A or ARHGDIB attenuated the increased migration and invasion caused by FOXA1 knockdown (Fig. 7k-o and S7i-k). The correlations between FOXA1 and KDM6A and between FOXA1 and ARHGDIB were then analysed using BCa specimens. As shown in Fig. 7p and q, the expression between FOXA1 and KDM6A was positively correlated, as was that between FOXA1 and ARHGDIB. A previous study verified that FOXA1 is a target gene of EZH2 [36]; however, neither overexpression nor knockdown of KDM6A changed the levels of FOXA1 in T24 cells (Fig. S7l). Taken together, these data demonstrate that the KDM6A-ARHGDIB axis is required for FOXA1 to inhibit migration and invasion in BCa cells.

## Discussion

Metastasis is the primary cause of cancer mortality. Therefore, the elucidation of the mechanisms that drive metastasis is crucial for the diagnosis, treatment, and prognosis of cancers. Acquisition of inappropriate migratory and invasive characteristics promoting metastasis is one of the hallmarks of cancer [37]. Dynamic reorganization of the actin cytoskeleton alters cell elongation and motility, thus increasing migration [38]. The abnormal expression or mutation of the proteins belonging to/interacting with the cell cytoskeleton enables cells to acquire an invasive and metastatic phenotype, which contributes to cancer progression and malignancy [39]. Rho GTPases are a family of highly conserved GTPases that regulate a range of fundamental cellular functions [40]. To date, the best characterized Rho GTPase functions involve modulating the actin cytoskeleton [41]. Rho GTPases have been reported to be upregulated or mutated in human cancers and have been shown to be crucial for tumour migration and invasion [40, 42, 43]. Therefore, the key components in this pathway are attractive targets for therapeutic interventions in cancer. ARHGDIB, a GDI that prevents the dissociation of bound GDP from the partner GTPases and inhibits Rho GTPase activation, was reported to bind Rac1 with high affinity [27]. ARHGDIB has been regarded as a metastasis suppressor in BCa. ARHGDIB expression is inversely associated with metastatic status and identified as an independent prognostic marker of tumour recurrence in BCa patients [26]. In addition, ARHGDIB expression is inversely correlated with the invasive and metastatic phenotype of BCa cell lines, and the upregulation of ARHGDIB suppresses cell invasion, motility, and lung metastasis in BCa cells [44]. We found that KDM6A decreased the active form of Rac1 and the F-actin content by upregulating ARHGDIB, and treatment with MBQ-167 could partly reverse the increased filopodia formation and invasive ability conferred by KDM6A knockdown, suggesting that KDM6A is involved in the cytoskeletal remodelling of BCa cells by regulating the ARHGDIB-Rac1 axis; additionally, targeting the ARHGDIB-Rac1 axis might have a potent effect on tumours with mutated or low expression of KDM6A in BCa patients.

Tumour-associated macrophages (TAMs) are a major component of the TME. Cancer cells secrete cytokines and chemokines to recruit monocytes to infiltrate cancer tissues and further promote the M2-type polarization. M2-like TAMs can in turn accelerate tumour growth, promote metastasis, and inhibit immune killing to promote tumour progression [45]. High TAM infiltration is correlated with poor clinical outcomes and decreases responses to standard-of-care therapeutics in BCa [22, 46]. We demonstrated that in BCa cells, KDM6A inhibits the recruitment of macrophages and the expression of IL-6 and CCL2, which are important for TAM recruitment [21]. These results, together with the finding that KDM6A is involved in “chemokine activity” and “cytokine-cytokine receptor interaction” from the transcriptome analysis, suggest that KDM6A plays an important role in TAM recruitment. This outcome can explain, at least in part, why KDM6A inhibits T24 tumour growth in vivo but does not affect the monolayer growth of T24 cells in vitro. These findings are consistent with a recent study, which found that KDM6A deficiency in the urothelium of mice activated the cytokine and chemokine pathways and promoted CCL2 and IL-6 expression and migration and M2 polarization in macrophages, contributing to the development of BCa [22]. Interestingly, ARHGDIB has been shown to inhibit the secretion of IL-6 and CCL2 and macrophage chemotaxis of human BCa cells [47]. Thus, the role of ARHGDIB must be fully understood to characterize the mechanisms through which KDM6A inhibits the recruitment of macrophages.

KDM6A has been shown to regulate gene transcription through both demethylase-dependent and demethylase-independent mechanisms [7]. Our results demonstrated that KDM6A acts as a histone methyltransferase to transactivate the ARHGDIB promoter in BCa cells. EZH2 antagonizes KDM6A by adding a methyl group to H3K27. The epigenetic imbalance between KDM6A and EZH2 has been observed in several cancer types due to loss-of-function mutation of KDM6A or gain-of-function mutation of EZH2 [48]. It has been reported that inhibition of EZH2 is more efficacious in cells and mice with KDM6A inactivating mutations, which creates EZH2 dependency in BCa cell proliferation [32]. Here, we found that EZH2 inhibited ARHGDIB expression. Treatment with the EZH2 inhibitor GSK126 decreased the amount of Rac1-GTP and neutralized the metastatic effect of KDM6A knockdown in vivo. These results indicate that BCa metastasis is mediated by the balance between KDM6A and EZH2 via the regulation of ARHGDIB expression. Thus, although an EZH2 inhibitor showed a limited effect on proliferation in KDM6A-wild-type cells [32], EZH2 inhibition could be regarded as a potential therapeutic process for suppressing the metastasis of BCa, regardless of the mutation status of KDM6A.

FOXA1 is a pioneer transcription factor that regulates cancer progression and differentiation, including liver, bladder, prostate, and lung cancers [49]. Several studies have reported that FOXA1 is a luminal marker and prognostic biomarker in BCa [50, 51], and a lack of nuclear expression for FOXA1 could be regarded as basal-like MIBC [52]. Continuous expression of FOXA1 can lead to a marked reduction in urothelial proliferation [53]. However, the detailed mechanisms of FOXA1 in regulating BCa metastasis are still not fully understood. We demonstrated that FOXA1 could bind to the promoter region of KDM6A and activate the transcription of KDM6A, consequently promoting ARHGDIB expression and inhibiting the migration and invasion of BCa cells, indicating that FOXA1 inhibits BCa metastasis by upregulating the KDM6A-ARHGDIB axis.

## Conclusion

**Fig. 8:**
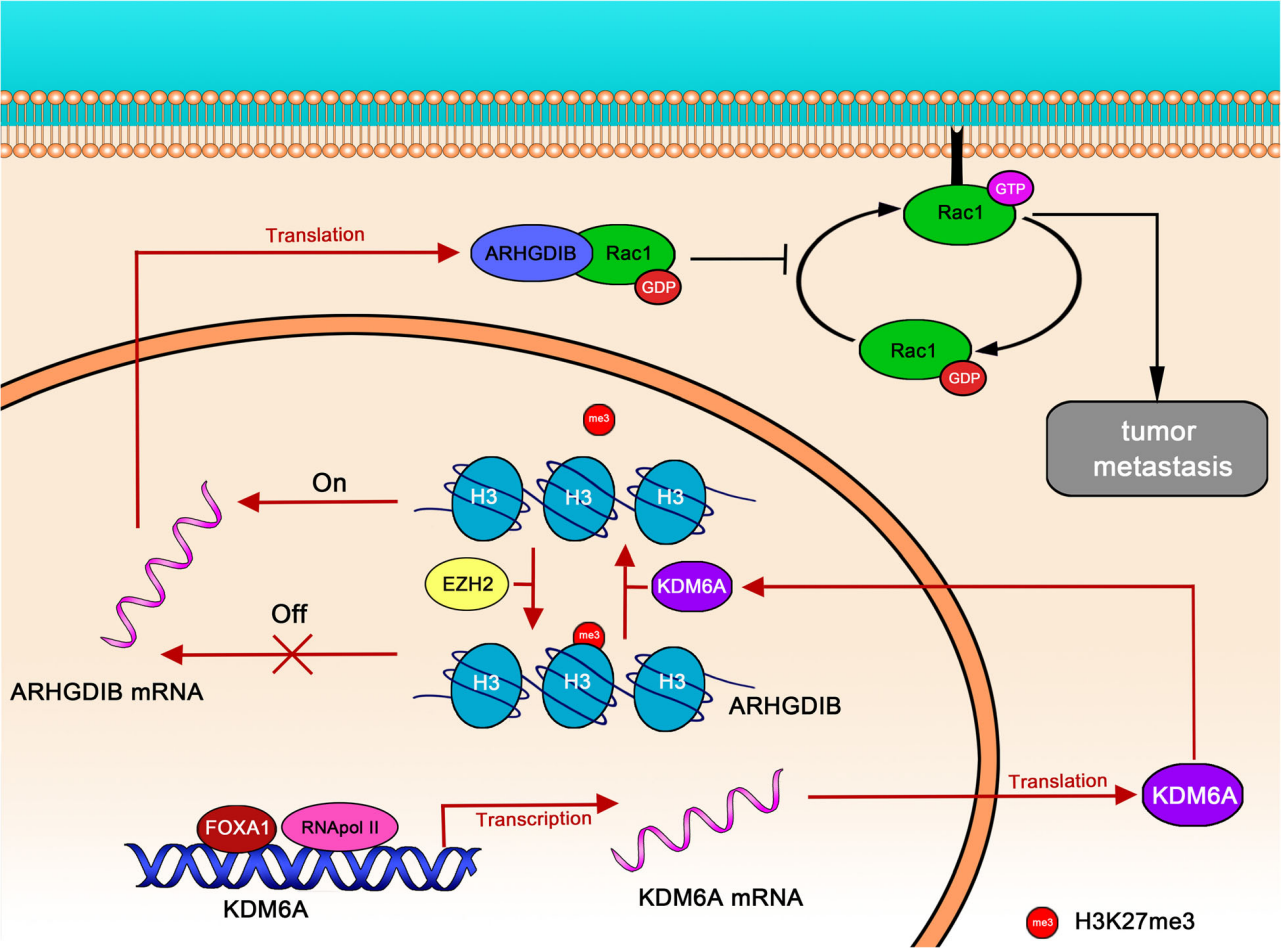
A schematic model showing FOXA1-KDM6A-ARHGDIB-Rac1 axis regulating BCa metastasis. FOXA1 binds to KDM6A promoter region and promotes its transcription. KDM6A can directly target the promoter region of ARHGDIB and promotes ARHGDIB transcription via demethylation of H3K27me3, antagonizing EHZ2 activity, and consequently inhibits the transformation from Rac1-GDP to active Rac1-GTP and therefore inhibits tumour metastasis

In conclusion, we described the role of the FOXA1-KDM6A-ARHGDIB axis in BCa. KDM6A is transcriptionally activated by FOXA1 and suppresses the metastasis of BCa cells via demethylation at H3K27 at the AHRGDIB promoter, leading to the upregulation of ARHGDIB and inactivation of Rac1. Moreover, EZH2 blocks KDM6A-dependent ARHGDIB transactivation, suggesting that ARHGDIB is regulated by the balance of KDM6A and EZH2 (Fig. 8). The precise mechanisms that underlie the antagonistic effect between KDM6A and EZH2 could be further assessed to develop therapeutic strategies for individual patients. Thus, future work focusing on the statuses of KDM6A and ARHGDIB could be useful in guiding clinical therapeutic decisions.

## Supplementary Information

**Additional file 1:** Supplementary Figures and Tables.

**Additional file 2.**

**Additional file 3.**

## Data Availability

All data needed to evaluate the conclusions in the paper are present in the paper and/or the Supplementary Materials. Additional data related to this paper may be requested from the authors.

## Abbreviations

BCa: Bladder cancer
H3K27me2/3: Histone H3 lysine di/trimethylation
NMIBC: Non-muscle-invasive bladder cancer
MIBC: Muscle-invasive bladder cancer
CIS: Carcinoma in situ
TP53: Tumour protein p53
FGFR3: Fibroblast growth factor receptor 3
PIK3CA: Phosphatidylinositol-4,5-bisphosphate 3-kinase catalytic subunit alpha
PTMs: Posttranslational modifications
KDM6A: Lysine Demethylase 6A
UTX: Ubiquitously transcribed tetratricopeptide repeat on chromosome X
T-ALL: T-cell acute lymphoblastic leukaemia
TAL1: TAL bHLH transcription factor 1
ARHGDIB: Rho GDP dissociation inhibitor beta
Rac1: Rac family small GTPase 1
FOXA1: Forkhead box A1
USF1: Upstream transcription factor 1
USF2: Upstream transcription factor 2
TFE3: Transcription factor binding to IGHM enhancer 3
EZH2: Enhancer of zeste 2 polycomb repressive complex 2 subunit
qPCR: real-time quantitative PCR
ChIP: Chromatin immunoprecipitation
MTT: 3-(4,5-Dimethylthiazol-2-yl)-2,5-diphenyl tetrazolium bromide
DMSO: Dimethyl sulfoxide
OD: optical density
IHC: Immunohistochemistry
BMDM: Bone marrow monocyte-derived macrophage
ELISA: Enzyme linked immunosorbent assay
IL-6: Interleukin-6
CCL2: C-C motif chemokine ligand 2
RNA-seq: RNA-sequencing
FDR: False discovery rate
DEG: Differentially expressed gene
GO: Gene Ontology
KEGG: Kyoto Encyclopaedia of Genes and Genomes
SD: Standard deviation
SEM: Standard error of the mean
TME: Tumour microenvironment
EMT: Epithelial-mesenchymal transition
MMP: Matrix metalloproteinase
OS: Overall survival
TCGA: The Cancer Genome Atlas
GDP: Guanosine diphosphate
GDI: GDP dissociation inhibitor
ARHGDIA: Rho GDP dissociation inhibitor alpha
ARHGDIG: Rho GDP dissociation inhibitor gamma
DOCK: Dedicator of cytokinesis
PRC2: Polycomb Repressive Complex 2
H3K4me1: monomethylated H3K4
H3K27ac: acetylated H3K27
IGFBP3: Insulin like growth factor binding protein 3
CDKN2A: Cyclin dependent kinase inhibitor 2A
TAM: Tumour-associated macrophage
