# Supplementary material for: KDM6A-ARHGDIB axis blocks metastasis of bladder cancer by inhibiting Rac1
Source: Mol Cancer. 2021 May 18;20:77. doi: 10.1186/s12943-021-01369-9 (PMC8130406; doi:10.1186/s12943-021-01369-9)
Supplement: Supplementary file 1 — Additional file 1: Supplementary Figures and Tables. [file 12943_2021_1369_MOESM1_ESM.docx]

**Supplementary material**





**Fig. S1 The role of KDM6A in proliferation of BCa cells.**

**a** The mRNA levels of KDM6A in BCa cells were determined by qPCR, SV-HUC-1 was set as 1. **b** DNA sequencing of T24 genomic DNA and reversed RNA. **c and d** The mRNA levels of KDM6A in RT4, T24 and 5637 cells were examined by qPCR (**c**, Puro was set as 1; D, NC was set as 1). **e and f** Effect of KDM6A knockdown on cell proliferation was examined by MTT **(e)** and colony formation assays **(f)** in RT4, T24 and 5637 cells. **g and h** Adenovirus mediated KDM6A overexpression in T24 and 5637 cells was verified by Western blot **(g)** and qPCR (**h**, GFP was set as 1). **i** MTT assays of the effect of KDM6A overexpression mediated by adenovirus on cell proliferation. **j and k** Growth curves of subcutaneous tumours formed by Puro/KDM6A **(j)** and NC/shKDM6A **(k)** T24 cells were shown. Tumour volumes were measured every 4 days. All quantification analyses were based on independent triplicate experiments. Error bars represent SD. **l and m** The weights of subcutaneous Puro/KDM6A **(l)** and NC/shKDM6A **(m)** T24 tumours were measured, Error bars represent SEM. **n** Representative images of macrophage chemotaxis assays of T24 Puro/KDM6A and NC/shKDM6A cells. **o** Representative IHC images of F4/80 in subcutaneous tumours were shown. Scale bars, 50 μm. **p and q** The IL-6 **(p)** and CCL2 **(q)** levels in culture supernatant from indicated T24 cells were determined by ELISA assays. **r and s** The mRNA levels of IL-6 **(r)** and CCL2 **(s)** in indicated T24 cells were detected by qPCR (WT was set as 1, KDM6A vs Puro, shKDM6A vs NC). All quantification analyses were based on independent triplicate experiments. Error bars represent SD. **p < 0.01, ***p < 0.001, NS no significant, based on Student’s t test.





**Fig. S2 KDM6A inhibits BCa cell migration and invasion.**

**a-c** Representative images of wound-healing assays **(a)**, Transwell migration assays **(b)**, and Transwell invasion assays **(c)** of indicated cells were shown. **d-f** Effect of KDM6A overexpression mediated by adenovirus on migration capacity in T24 and 5637 cells was tested by wound healing assays **(d-e)** and Transwell invasion assays **(f)**. **g-i** Representative images of wound-healing assays **(g)**, Transwell migration **(h)** and Matrigel invasion assays **(i)** of indicated cells were shown. **j and k** The mRNA levels of E-Cadherin (E-Cad), N-Cadherin (N-Cad), Snail, Zeb1 and Vimentin in indicated T24 **(j)** and 5637 **(k)** cells were examined by qPCR (WT was set as 1, KDM6A vs Puro, shKDM6A vs NC). **l** The mRNA levels of MMP1, MMP2 and MMP9 in indicated T24 cells were detected by qPCR (WT was set as 1, KDM6A vs Puro, shKDM6A vs NC). **m and n** Cell viability of Puro/KDM6A **(m)** and NC/shKDM6A **(n)** T24 cells treated with CDDP for 2 days were measured by MTT. The normalized O.D value of control cells at day 0 was set as 1. **o** Representative IHC images of KDM6A in tissue microarray were shown. Scale bar, 500 μm. **p** Kaplan-Meier survival curve of BCa patients based on KDM6A mRNA expression levels from TCGA database. All quantification analyses were based on independent triplicate experiments. Error bars represent SD. Scale bar 50 μm. *p<0.05, **p < 0.01, ***p < 0.001, NS no significant, based on Student’s t test.

**Fig. S3 KDM6A promotes ARHGDIB transcription in BCa cells.**





**a** Volcano plot of genes differentially expressed (Fold change>1.5, false discovery rate<0.05) in KDM6A vs Puro and shKDM6A vs NC T24 cells by RNA-seq. Significantly up-regulated genes are represented as ‘red’ dots, significant down-regulated genes are represented as ‘green’ dots in volcano plot and unchanged genes are represented as ‘blue’ dots. **b and c** qPCR analysis of the levels of 42 genes in KDM6A/Puro and shKDM6A/NC T24 cells, including 21 genes upregulated in KDM6A overexpressed group and downregulated in shKDM6A group **(b)**, as well as 21 genes downregulated in KDM6A overexpressed group and upregulated in shKDM6A group **(c)** according to RNA-seq. **d and e** The mRNA levels of ARHGDIA **(d)** and ARHGDIG **(e)** in indicated T24 cells were detected by qPCR (WT was set as 1; KDM6A vs Puro, shKDM6A vs NC). **f** The effects of KDM6A overexpression mediated by adenovirus on ARHGDIB expression levels were detected by Western blot in indicated cells. All quantification analyses were based on independent triplicate experiments. NS no significant, based on Student’s t test.





**Fig. S4 ARHGDIB acts as a downstream effector of KDM6A to mediate cell migration and invasion inhibition.**

**a** Representative images of wound healing assays of siNC/siARHGDIB T24 cells were shown. **b and c** Representative images of Transwell migration **(b)** and Matrigel invasion **(c)** assays of siNC/siARHGDIB T24 cells were shown. **d** Kaplan-Meier survival curve of the patients with BCa based on ARHGDIB mRNA levels from TCGA database. **e and f** Representative images of wound healing assays **(e)** and Transwell invasion assays **(f)** of indicated cells were shown. **g and h** Representative images of macrophage chemotaxis assays of bone marrow monocyte-derived macrophages (BMDMs) cultured with conditioned medium from indicated cells. **i-l** The correlation between clinicopathologic parameters and KDM6A/ARHGDIB. KDM6A/T stage **(i)**, KDM6A/Clinical stage **(j)**, ARHGDIB/T stage **(k)**, ARHGDIB /Clinical stage **(l)**, based on the chi-square test. All quantification analyses were based on independent triplicate experiments. Scale bar 50 μm.


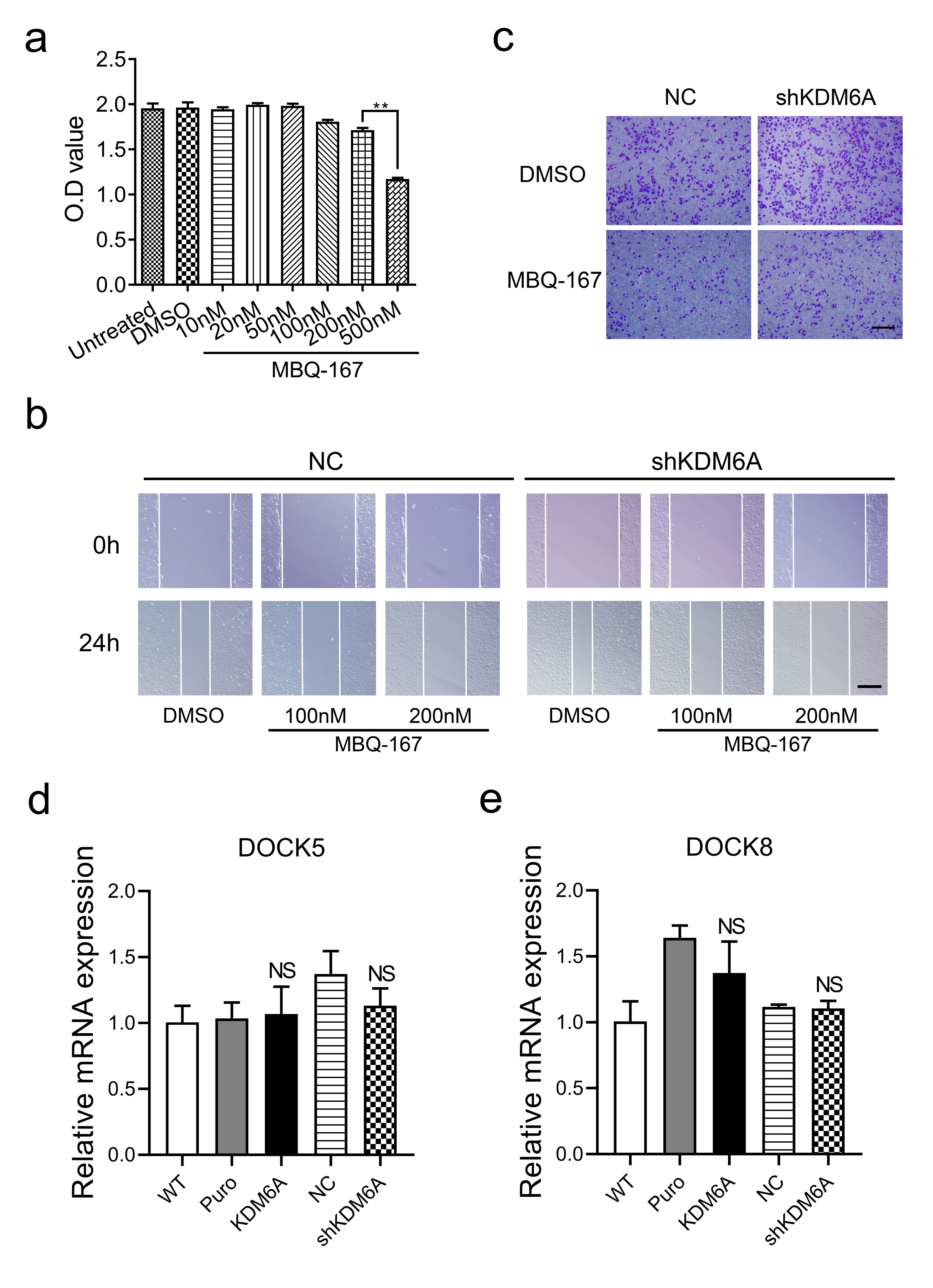


**Fig. S5 KDM6A suppresses BCa cell migration and invasion via Rac1 inhibition.**

**a** The cell viability of T24 cells treated with MBQ-167 for 48 h was assessed by MTT assays. **b and c** Representative images of wound healing assays **(b)** and Transwell invasion assays **(c)** of NC/shKDM6A T24 cells treated with MBQ-167 were shown. **d and e** The mRNA levels of DOCK5 **(d)** and DOCK8 **(e)** in indicated T24 cells were detected by qPCR (WT was set as 1, KDM6A vs Puro, shKDM6A vs NC). All quantification analyses were based on independent triplicate experiments. Error bars represent SD. Scale bar 50 μm. **p < 0.01, NS no significant, based on Student’s t test.





**Fig. S6 KDM6A promotes ARHGDIB transcription by catalyzing demethylation of H3K27me3.**

**a** The nuclear and cytoplasmic KDM6A protein levels in indicated cells were assessed by Western blot. **b** Indicated protein levels in Puro/KDM6A and NC/shKDM6A 5637 cells were detected by Western blot. **c** EZH2 protein levels in indicated cells were detected by Western blot. **d-i** The mRNA levels of ARHGDIB in indicated cells treated with GSK126 for 48 h were measured by qPCR (DMSO was set as 1), 5637 **(d)**, SV-HUC-1 **(e)**, HeLa **(f)**, MKN-45**(g)**, A549 **(h)**, U2OS **(i)**. **j** The representative images of mice lungs in indicated groups were shown. **k** The mRNA levels of IGFBP3 in indicated T24 cells were detected by qPCR (WT was set as 1; KDM6A vs Puro, shKDM6A vs NC). **l** The mRNA levels of CDKN2A in indicated T24 cells were detected by qPCR (WT was set as 1; KDM6A vs Puro, shKDM6A vs NC). All quantification analysis was based on independent triplicate experiments. Error bars represent SD. *p<0.05, **p < 0.01, ***p < 0.001, NS no significant, based on Student’s t test.


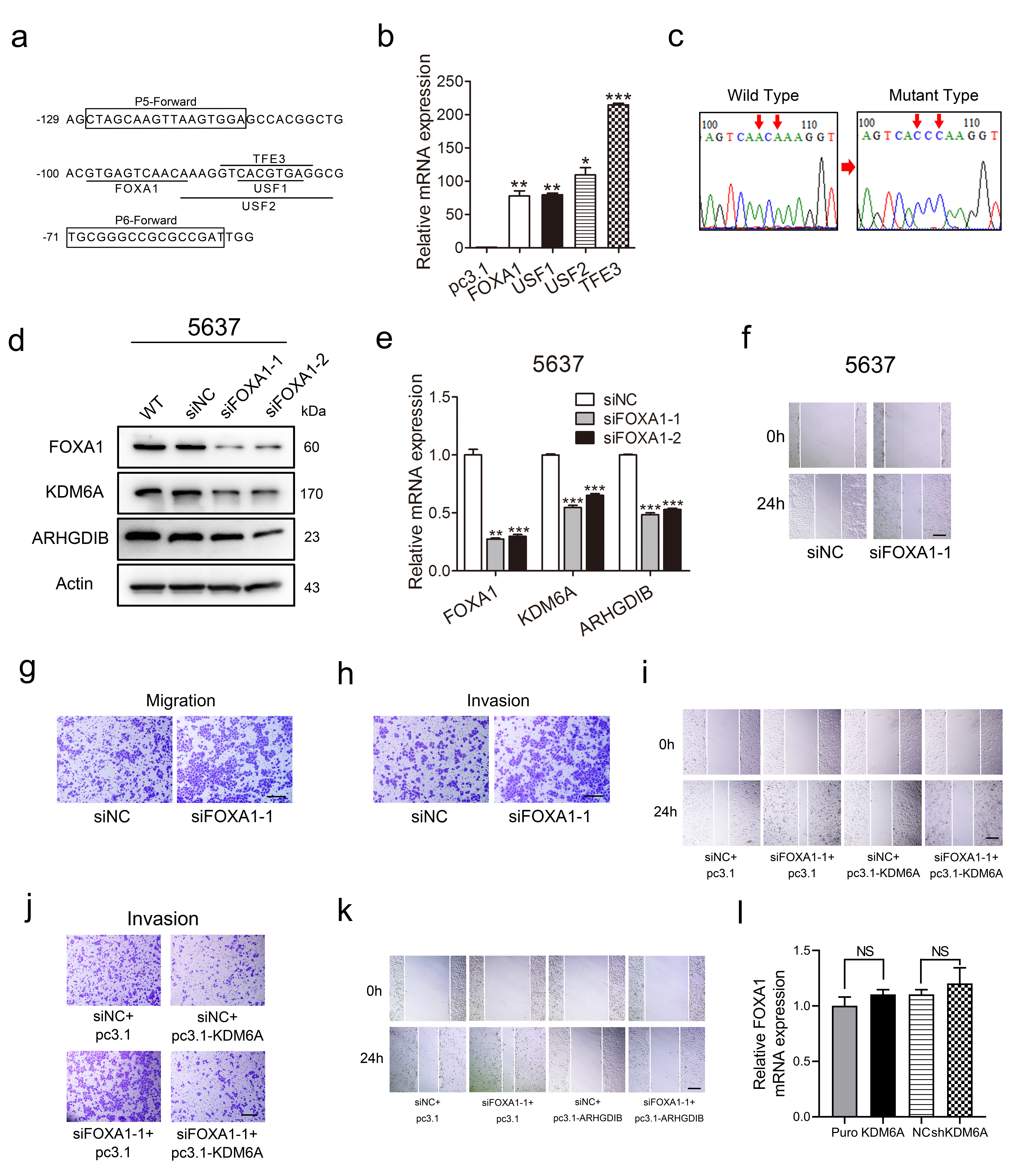


**Fig. S7 FOXA1 binds to KDM6A promoter and active KDM6A transcription.**

**a** Schematic diagram showed FOXA1, USF1, USF2 and TFE3 binding sites in KDM6A promoter regions predicted by JASPAR. **b** Transient transfection mediated FOXA1, USF1, USF2 and TFE3 overexpression in T24 was verified by qPCR (pc3.1 was set as 1). **c** Point mutations in predicted FOXA1 binding site were verified by DNA sequencing. **d and e** KDM6A and ARHGDIB expression in indicated 5637 cells was detected by Western blot **(d)** and qPCR (**e**; siNC was set as 1). **f-h** Representative images of wound healing assays **(f)**, Transwell migration assays **(g)** and Transwell invasion assays **(h)** of indicated T24 cells were shown. **i-j** Representative images of wound healing assays **(i)** and Transwell invasion assays **(j)** of indicated T24 cells were shown. **k** Representative images of wound healing assays of indicated T24 cells were shown. **l** The mRNA levels of FOXA1 in indicated T24 cells were detected by qPCR (Puro was set as 1; KDM6A vs Puro, shKDM6A vs NC). All quantification analyses were based on independent triplicate experiments. Error bars represent SD. Scale bar 50 μm. **p < 0.01, ***p < 0.001, NS no significant, based on Student’s t test.

**Table S1 Characteristics and gene mutations in bladder cancer cell lines**

| Name | Gender | Year | Source | Grade | Mutant gene | TP53  (Mutation/CN) | INK4A status | Losses of CNV in chromosome | Gains of CNV in chromosome | Database | Reference |
| --- | --- | --- | --- | --- | --- | --- | --- | --- | --- | --- | --- |
| RT4 | Male | 63 | Bladder | G1 | CDKN2A, TSC1, TERT, | WT/LOH | HD | 9, 10, 13, 16, 17p |  | ATCC, COSMIC, IARC | [1-3] |
| T24 | Female | 81 | Bladder | G3 | HRAS, TP53, TERT, | c.839G>C/N | WT | 8p, 9p, 12, 18 | 20, 22 | ATCC, COSMIC, IARC, CCLE | [1-3] |
| 5637 | Male | 68 | Bladder | G2 | TP53, RB1, TERT, | c.378C>G/N | WT/LOH | 3p, 4, 6p, 8p, 10, 16, 18q | 5p, 7, 9p, 20q | ATCC, COSMIC, IARC | [1, 2, 4] |
| CN = Copy number, CNV = Copy number variation, WT = Wild type, LOH = Loss of heterozygosity, HD = Homozygous deletion, N = Copy number neutral  Reference  [1] Hurst CD, Platt FM, Knowles MA. Comprehensive mutation analysis of the TERT promoter in bladder cancer and detection of mutations in voided urine. Eur Urol. 2014, 65(2):367-369.  [2] Earl J, Rico D, Carrillo-de-Santa-Pau E, Rodríguez-Santiago B, Méndez-Pertuz M, Auer H, Gómez G, Grossman HB, Pisano DG, Schulz WA, et al. The UBC-40 Urothelial Bladder Cancer cell line index: a genomic resource for functional studies. BMC Genomics. 2015, 16(1):403.  [3] Masters JR, Hepburn PJ, Walker L, Highman WJ, Trejdosiewicz LK, Povey S, Parkar M, Hill BT, Riddle PR, Franks LM. Tissue Culture Model of Transitional Cell Carcinoma: Characterization of Twenty-two Human Urothelial Cell Lines. Cancer Res. 1986, 46(7):3630-6.  [4] Fogh J, Wright WC, Loveless JD. Absence of HeLa cell contamination in 169 cell lines derived from human tumors. J Natl Cancer Inst. 1977, 58(2):209-14. | | | | | | | | | | | |

**Table S2 Primers used for construction of overexpression vectors**

| Vector | Primers | Sequences |
| --- | --- | --- |
| pLVX-IRES-Puro-Mutant KDM6A | Forward | CTACTCGAGATGAAATCCTGCGGAGTGTC |
|  | Reverse | CGGTCTAGATCAAGATGAGGCGGATGGT |
| pcDNA3.1(+)/Myc-His B-FOXA1 | Forward | CGGAATTCATGTTAGGAACTGTGAAGATGG |
|  | Reverse | GCTCTAGATAGGAAGTGTTTAGGACGGG |
| pcDNA3.1(+)/Myc-His B-ARHGDIB | Forward | CGGAATTCATGACTGAAAAAGCCCCAGA |
|  | Reverse | GCTCTAGATCATTCTGTCCACTCCTTCTTAATC |
| pcDNA3.1(+)/Myc-His B-USF1 | Forward | CGGAATTCATGAAGGGGCAGCAGAAAACA |
|  | Reverse | GCTCTAGATAGTTGCTGTCATTCTTGAT |
| pcDNA3.1(+)/Myc-His B-USF2 | Forward | CGGAATTCATGGACATGCTGGACC |
|  | Reverse | GCTCTAGACACTGCCGGGTGCCCTCG |
| pcDNA3.1(+)/Myc-His B-TFE3 | Reverse | GTGAAGCTTATGTCTCATGCGGCCGAAC |
|  | Reverse | GCTCTAGACAGGACTCCTCTTCCATGC |

**Table S3 RT-qPCR primers**

| Gene name | Primer | Sequences | Product length（bp） |
| --- | --- | --- | --- |
| ABLIM1 | Forward | TGAGCCTTTCTACACTTCGGG | 93 |
|  | Reverse | TCTGCTGATGGAGTAGGAGAC |  |
| AKR1C1 | Forward | TCCAGTGTCTGTAAAGCCAGG | 257 |
|  | Reverse | CCAGCAGTTTTCTCTGGTTGAA |  |
| AKR1C3 | Forward | GAGACAAACGATGGGTGGACC | 193 |
|  | Reverse | TGGAACTCAAAAACCTGCACG |  |
| ANGPTL4 | Forward | GTCCACCGACCTCCCGTTA | 212 |
|  | Reverse | CCTCATGGTCTAGGTGCTTGT |  |
| ANKRD1 | Forward | GCCTACGTTTCTGAAGGCTG | 201 |
|  | Reverse | GTGGATTCAAGCATATCACGGAA |  |
| AQP11 | Forward | GCTCAAAGCGGTCATCACAGA | 102 |
|  | Reverse | GCCAGCAGGTGGATACGAAG |  |
| ARHGDIA | Forward | GGATGAGCACTCGGTCAACTA | 103 |
|  | Reverse | GGCCTCCTTGTACTTTCGCAG |  |
| ARHGDIB | Forward | GACTGGGGTGAAAGTGGATAAAG | 150 |
|  | Reverse | TCGTCGGTGAAGAAGGACTTG |  |
| ARHGDIG | Forward | GAGTTTGTGACTCCGGTGGA | 85 |
|  | Reverse | CATCGTCGGTGAAGAGGGAC |  |
| BACE2 | Forward | AGACACGTACTTTGACACAGAGA | 122 |
|  | Reverse | TGGGGATGGTGACGAGGTC |  |
| BAMBI | Forward | AGCACGACAGACATCTGCC | 230 |
|  | Reverse | CGGAACCACAACTCTTTGGAAG |  |
| CAP1 | Forward | GGGGAGACGTGCAGAAACAT | 125 |
|  | Reverse | GGGTGCCAACAAATCGGAAAG |  |
| CCL2 | Forward | AATCAATGCCCCAGTCACCT | 107 |
|  | Reverse | CTTCTTTGGGACACTTGCTGC |  |
| CD164 | Forward | AGCCAATTCTACAGCTAAACCC | 184 |
|  | Reverse | AATTACAGCCTGCACACCCAA |  |
| CHD7 | Forward | AGGCCGCAAACCCTTAACTTT | 179 |
|  | Reverse | GGTACGGACTGATTCATTGGAG |  |
| CLMP | Forward | GAATGGCTGCTCACCGATAAT | 158 |
|  | Reverse | TTCAGAGGTTCAATCTGCAAGG |  |
| CTGF | Forward | CAGCATGGACGTTCGTCTG | 115 |
|  | Reverse | AACCACGGTTTGGTCCTTGG |  |
| CXCL5 | Forward | AGCTGCGTTGCGTTTGTTTAC | 75 |
|  | Reverse | TGGCGAACACTTGCAGATTAC |  |
| DAP3 | Forward | TCCAGCTACAACAAACAGCG | 191 |
|  | Reverse | CTCACCCGTGTTATGCCCTG |  |
| E2F8 | Forward | ATCTGCCTTGACGAAGTGGC | 189 |
|  | Reverse | GGCGTACTTATTCTCCTCCCC |  |
| ELAC2 | Forward | AAACCGGGCTTCCAAAGTGT | 169 |
|  | Reverse | TGTATGGGGATCTGGTAAACTGT |  |
| ERRFI1 | Forward | GACCCACCGAAGATTAAGAAGG | 156 |
|  | Reverse | GGTCTAGGAGGTATGGGAACTCT |  |
| EZH2 | Forward | AATCAGAGTACATGCGACTGAGA | 141 |
|  | Reverse | GCTGTATCCTTCGCTGTTTCC |  |
| FOXA1 | Forward | GCAATACTCGCCTTACGGCT | 120 |
|  | Reverse | TACACACCTTGGTAGTACGCC |  |
| FXR1 | Forward | GAGAAGACGGTATGGTTCCATTT | 124 |
|  | Reverse | AGGCGTTCCATTCTTAGCTGT |  |
| GAPDH | Forward | CCCACTCCTCCACCTTTGAC | 115 |
|  | Reverse | ATGAGGTCCACCACCCTGTT |  |
| GIPC1 | Forward | TTCTGCACCCTGAACACCCAC | 131 |
|  | Reverse | GCATCCTCCGACTTGAACACCT |  |
| GNAL | Forward | CACGTCAATGGGTTTAATCCCG | 187 |
|  | Reverse | CAAAGTCAGTGATAGGGGCTATG |  |
| H2AFY2 | Forward | GCCCCGAGACACATCTTGC | 78 |
|  | Reverse | ACTGGCGATGGTCACTCCT |  |
| HCLS1 | Forward | CTATGGAGGTCGGTTTGGAGT | 104 |
|  | Reverse | CAGCATCCGTCTGAGAAGAGT |  |
| HNRNPL | Forward | TCGATCACCACGGATGTTCTT | 206 |
|  | Reverse | AAGCGTGTAGGCTTTGCGT |  |
| IL-6 | Forward | CAATGAGGAGACTTGCCTGGT | 146 |
|  | Reverse | GCAGGAACTGGATCAGGACT |  |
| KDM6A | Forward | TACAGGCTCAGTTGTGTAACCT | 99 |
|  | Reverse | CTGCGGGAATTGGTAGGCTC |  |
| LAMA3 | Forward | CACCGGGATATTTCGGGAATC | 165 |
|  | Reverse | AGCTGTCGCAATCATCACATT |  |
| LAMA4 | Forward | CCAGTGTAGGAATTGCTTACGC | 217 |
|  | Reverse | TAACCGCAGGTCATCAGTCAG |  |
| LIG4 | Forward | TGATCCTTCTGTAGGACTCAGTG | 158 |
|  | Reverse | GCATTTGCATACGTTCACCATC |  |
| MAGEA1 | Forward | GTGATCCCGCACGCTATGAG | 108 |
|  | Reverse | AAAAGCGAACTCTTGCACTGA |  |
| MBD4 | Forward | CCGTCACCTCTAGTGAGCG | 160 |
|  | Reverse | GCAGAAGCGATGGGTTCTTGTA |  |
| MTSS1 | Forward | CAGTCCCAGCTTCGGACAAC | 118 |
|  | Reverse | TGAGAGCAGATCCAATCTCCC |  |
| PCDHB12 | Forward | AAACGACAACGCTCCTGAAAT | 100 |
|  | Reverse | TCTGTCTCGTATCCTGAAAACCA |  |
| PDGFD | Forward | TTGTACCGAAGAGATGAGACCA | 133 |
|  | Reverse | GCTGTATCCGTGTATTCTCCTGA |  |
| POLL | Forward | GATGAAGGCATGGACTATGAGC | 136 |
|  | Reverse | TGAATCCAGCTACATCCACCAG |  |
| PSMC3 | Forward | CGAGCAAGATGGAATTGGGGA | 125 |
|  | Reverse | GCTCATGGGTGACTCTCAACA |  |
| RAB27B | Forward | TAGACTTTCGGGAAAAACGTGTG | 192 |
|  | Reverse | AGAAGCTCTGTTGACTGGTGA |  |
| RACGAP1 | Forward | TCCAATTTATCCAGTTGGCGAA | 137 |
|  | Reverse | CTTCAGCTTAACATCCAGAGCA |  |
| RORA | Forward | ACTCCTGTCCTCGTCAGAAGA | 190 |
|  | Reverse | CATCCCTACGGCAAGGCATTT |  |
| RPS15A | Forward | CTCCAAAGTCATCGTCCGGTT | 161 |
|  | Reverse | TGAGTTGCACGTCAAATCTGG |  |
| RPS6KA2 | Forward | GCCACCCTAAAAGTTCGGGAC | 113 |
|  | Reverse | TTTCCTTCCGTCTGAAAGGCA |  |
| S100A4 | Forward | GATGAGCAACTTGGACAGCAA | 123 |
|  | Reverse | CTGGGCTGCTTATCTGGGAAG |  |
| SCARA3 | Forward | TCACCCAGGAGTGCTACGAT | 158 |
|  | Reverse | GAGCCGTGTGTAGTTCTGCC |  |
| SGK1 | Forward | AGGATGGGTCTGAACGACTTT | 228 |
|  | Reverse | GCCCTTTCCGATCACTTTCAAG |  |
| SLC1A1 | Forward | GCGAGGAAAGGATGCGAGT | 114 |
|  | Reverse | GCTGTGTTCTCGAACCAAGACT |  |
| SLC8A1 | Forward | TCATAGCTGATCGGTTCATGTCC | 99 |
|  | Reverse | CAGTTGTCTTGGTGGTCTCTC |  |
| SMARCA2 | Forward | AGCGGGAATACAGACTTCAGG | 116 |
|  | Reverse | AAGTGCTTTTAGTTCCACGGTT |  |
| SOCS2 | Forward | TTAAAAGAGGCACCAGAAGGAAC | 199 |
|  | Reverse | AGTCGATCAGATGAACCACACT |  |
| SPNS2 | Forward | ATGGCTCCGAGATATGAAGGC | 146 |
|  | Reverse | CTGTCTTCTGCACAACTTGGG |  |
| STMN1 | Forward | TCAGCCCTCGGTCAAAAGAAT | 169 |
|  | Reverse | TTCTCGTGCTCTCGTTTCTCA |  |
| SUMO1 | Forward | TGACCAGGAGGCAAAACCTTC | 176 |
|  | Reverse | AATTCATTGGAACACCCTGTCTT |  |
| TFE3 | Forward | CCGTGTTCGTGCTGTTGGA | 138 |
|  | Reverse | GCTCGTAGAAGCTGTCAGGAT |  |
| TLE4 | Forward | CCCAGCATTTATCACATGGACA | 97 |
|  | Reverse | GCACTGCTACCGATGGGTG |  |
| TRIM22 | Forward | ACCAAACATTCCGCATAAACGA | 132 |
|  | Reverse | AGGCGGTTCTCTCTTGTCTGA |  |
| USF1 | Forward | CTGCTGTTGTTACTACCCAGG | 160 |
|  | Reverse | TCTGACTTCGGGGAATAAGGG |  |
| USF2 | Forward | CCCGGACACACCCTTACTCT | 87 |
|  | Reverse | GCTCCACTTCGTTGTGCTG |  |

**Table S4 Primers for FOXA1 binding KDM6A promoter**

| Name | Primers | Sequences | Product length（bp） |
| --- | --- | --- | --- |
| A | Forward | GAGTCTCGCTCTGTCACCC | 177 |
|  | Reverse | AGATGGAGACCACGGTGAAA |  |
| B | Forward | CGCCCAGCAGCCAACCACC | 173 |
|  | Reverse | CCCGGCTGGCGCCACCAATC |  |
| C | Forward | AATGGAAACGTGCCTTACCTG | 148 |
|  | Reverse | CTGCCGAATGTGAACTCTGACC |  |

**Table S5 Primers for KDM6A binding ARHGDIB promoter**

| Name | Primers | Sequences | Product length（bp） |
| --- | --- | --- | --- |
| 1 | Forward | TAGAATAAAGTGGTATTAGGC | 207 |
|  | Reverse | GTTTTCACCTAAATGTACTGC |  |
| 2 | Forward | GAAGATGCAGTACATTTAGGT | 238 |
|  | Reverse | TTTAAATGGGAACAAGTCTCG |  |
| 3 | Forward | TTAAACATGGCCTCCGAGA | 240 |
|  | Reverse | TTGTTTTTAATAGTTTTGGCT |  |
| 4 | Forward | CAGCCAAAACTATTAAAAACAAT | 211 |
|  | Reverse | CCAGCTTCATTATCAACCAG |  |
| 5 | Forward | TATCTGGTTGATAATGAAGCTG | 212 |
|  | Reverse | ATGCATGAATGTACACGCAAG |  |
| 6 | Forward | CGTGTACATTCATGCATCTC | 234 |
|  | Reverse | GCCTCTGTCTCTCAACTCT |  |
| 7 | Forward | AAGTCAGAGTTGAGAGACAGA | 223 |
|  | Reverse | AGCAGCGTTTTCTTGTACT |  |
| 8 | Forward | AATCTTGAGCTGCTTGCTT | 213 |
|  | Reverse | GTACATGTAAGGACATCAAGG |  |
| 9 | Forward | AAAGCCTTCCTTGATGTCC | 233 |
|  | Reverse | AGAAAGTTCTAAAAGGACTC |  |
| 10 | Forward | TATTTTGATAATTGGAGTCCT | 232 |
|  | Reverse | CCACCACTCATTTCTCATGT |  |
| 11 | Forward | GTGTTCCCTAATAACATGAGA | 232 |
|  | Reverse | CATAACATTTATCGAGCACCT |  |
| 12 | Forward | AAAGAAAAGAGGTGCTCGAT | 211 |

**Table S6 Primers for construction of KDM6A promoter vectors**

| Name | Primers | Sequences | Product length（bp） |
| --- | --- | --- | --- |
| P1 | Forward | GCCCTCGAGAGCGATTCTTCTGCCTCAGTC | 2115 |
| P2 | Forward | GCCCTCGAGCTCCACTGAAATCATGGCAA | 1045 |
| P3 | Forward | CTCTCGAGGGAGCCGCGGTCTGGGCAC | 528 |
| P4 | Forward | GCCCTCGAGAGCAGCCAACCACCGGCTA | 271 |
| P5 | Forward | GTCCTCGAGCTAGCAAGTTAAGTGGA | 192 |
| P6 | Forward | GTCCTCGAGTGCGGGCCGCGCCGAT | 136 |
| P1-6 | Reverse | CCCAAGCTTACAAACTTCCCCGGCACCA |  |
| mP5 | Forward | GGGAGCAGAACACCAGGTGCTCAGGCAAATAACAACTTCTGTTC |  |
|  | Reverse | GAACAGAAGTTGTTATTTGCCTGAGCACCTGGTGTTCTGCTCCC |  |
